# Unravelling the association between accelerometer‐derived physical activity and adiposity among preschool children: A systematic review and meta‐analyses

**DOI:** 10.1111/obr.12936

**Published:** 2019-12-13

**Authors:** Rikstje Wiersma, Barbara F. Haverkamp, Jasper H. van Beek, André M.J. Riemersma, H. Marike Boezen, Nynke Smidt, Eva Corpeleijn, Esther Hartman

**Affiliations:** ^1^ Department of Epidemiology, University Medical Center Groningen University of Groningen Groningen The Netherlands; ^2^ Center for Human Movement Sciences, Section F, University Medical Center Groningen University of Groningen Groningen The Netherlands; ^3^ Clinical Neuropsychology Section, Faculty of Behavioural and Movement Sciences Vrije Universiteit Amsterdam Amsterdam The Netherlands

**Keywords:** accelerometry, body fat distribution, exercise, sedentary behaviour

## Abstract

Evidence on the association between physical activity (PA) and adiposity in young children is inconclusive. A systematic review and meta‐analyses were conducted to examine associations between accelerometer‐derived PA and varying adiposity outcomes in preschool children. Searches were conducted in Embase, MEDLINE and Web of Science to identify studies on the association between total PA, sedentary behaviour or different PA intensities and adiposity in children aged 2 to 7 years. Separate random effects meta‐analyses were performed for varying PA intensities and adiposity outcomes. Fifty‐six articles were included in the review and 48 in the meta‐analyses. There was substantial evidence of an inverse association between moderate‐to‐vigorous‐ or vigorous PA and body fat percentage (stdβ [SE] = −0.162[0.041]; 5 studies), weight status (*r* = −0.120, *P*<.001; 11 studies), fat mass (stdβ [SE] = −0.103[0.051]; 5 studies), fat mass index (stdβ [SE] = −0.121[0.036]; 2 studies) and skinfold thickness (stdβ [SE] = −0.145[0.036]; 4 studies). However, total PA, sedentary behaviour, and different PA intensities were not associated with body mass index (BMI) or waist circumference. Adiposity levels were lower among preschool children engaged in more (moderate‐to‐) vigorous PA compared with their peers, but no associations between PA and BMI or waist circumference were found.

## 1 INTRODUCTION

Overweight and obesity is an increasing problem in society, in adulthood and in childhood. In 2016, about 6.0% of the under‐five age group, globally, were affected by overweight or obesity, with percentages ranging from 3.7% in Africa to 7.2% in the United States.^1^ During childhood, many children with obesity develop health problems that once emerged only in adults.^2^ Cardiometabolic, pulmonary and psychosocial complications as well as orthopaedic disorders, liver and gall bladder dysfunction, cardiovascular and endocrine problems and cancer are seen in children with obesity.^2^, ^3^ Furthermore, a lot of children with obesity will be affected by obesity in adulthood as well.^4^ Research on young children is important because prevention of overweight at young age is more effective compared with treatment after its onset.^3^, ^5^

Body weight increases when energy intake (nutrition) chronically exceeds total body energy expenditure. In children, this energy expenditure is the sum of physical activity (PA), growth, the basal metabolic rate and environment‐ and diet‐induced thermogenesis.^6^ Therefore, PA is seen as a key component in the prevention and management of obesity.^3^, ^7^, ^8^ However, while overweight is increasingly prevalent among preschool children, evidence of the association between PA and the development of overweight in this age group is inconclusive.

The use of different methods within studies conducted to assess the association between PA and adiposity has led to inconsistent findings. A review conducted in 2012 covered 17 studies that examined the relation between PA, sedentary behaviour and childhood obesity. The review revealed the use of six subjective and objective methods for assessing PA and reported mixed results.^9^ Whereas some of the studies found a negative association between PA and weight status, others found that there was no association between them.^9^ In another study, 48 studies on the association between PA and adiposity in children and adolescents were reviewed.^10^ Only studies in which PA was measured objectively were included, but the results remained dependent on the instrument used to measure PA. A negative association was found in all of the studies using pedometers (n = 11) and in 72% of the studies in which accelerometers were used (n = 32).^10^ One review focused solely on objectively measured PA and adiposity in preschool children.^11^ In this review, no clear association was found between objectively measured PA and weight status. The authors suggest that the association between objectively measured PA and weight status in preschool children depends on the outcome measures used.^11^

Several methods have been used to assess young children's PA. Measurements taken with accelerometers are reliable and valid and enable differences in frequency, duration and PA intensity to be objectively assessed.^12^, ^13^ These measurements are extremely important in the case of preschool children.^14^ Pedometers also provide objective measurements. However, the possibilities to assess differences in intensities and movement directions with these instruments are scarce. Questionnaires provide subjective responses, and their reliability and validity are limited, especially when used for young children.^15^ Thus, whereas accelerometers also have limitations, they are potentially the most effective instruments for assessing PA in preschool children.

In the last few years, more studies measuring PA using accelerometers have been conducted. Therefore, this review focused exclusively on these studies, thereby reducing differences caused by the use of various instruments for measuring PA. Furthermore, it is possible to distinguish between different PA intensities and to conduct meta‐analyses to explore heterogeneous results. The aim of the current study was to conduct a review and meta‐analyses to examine whether accelerometer‐derived PA is related to adiposity at preschool ages. We anticipated varying associations for different PA intensities and different adiposity outcomes. Hence, we differentiated PA intensities(total PA, sedentary behaviour, light PA, moderate PA, vigorous PA and moderate‐to‐vigorous PA [MVPA]) and different adiposity outcomes (percentage body fat, body mass index [BMI], weight status, waist circumference, fat mass and skinfold thickness). The differentiation of PA intensities and adiposity outcomes can provide researchers and policymakers with directly applicable information enabling the targeting of PA intensities to prevent increases in childhood overweight/obesity and the determination of which adiposity outcomes are related to PA.

## 2 METHODS

We registered the protocol of this systematic review and meta‐analyses in the International Prospective Register of Systematic Reviews (PROSPERO, http://www.crd.york.ac.uk/PROSPERO/ display_record.php?ID=CRD42018082660) and adhered to the methods of the Cochrane Collaboration. We followed Preferred Reporting Items for Systematic Reviews and Meta‐Analyses (PRISMA) guidelines for reporting the findings.^16^

### 2.1 Search strategy

Relevant studies for the systematic review were identified through a literature search in Embase, MEDLINE and Web of Science. A combination of MeSH terms and keywords were used: (children or preschool or paediatrics) and (“body mass index” or BMI or “body fat” or “waist circumference” or overweight or obese or “weight status” or “body composition”) and (“physical activity” or exercise or “activity level” or “sedentary behaviour”) and (accelerometry or “physiologic monitoring” or actigraph) (Appendix A). The literature search was performed on 13 August 2018. Additional studies were identified by searching the reference lists of the included studies. All identified hits (for abstracts and titles) were screened for eligibility by two independent reviewers (R. W. and J. v. B.). Subsequently, the same two reviewers independently read the full text of all potentially eligible papers. Disagreements were discussed during a consensus meeting, and in case of persistent disagreement, a third reviewer (B. H.) was consulted.

### 2.2 Inclusion and exclusion criteria

Studies that met the following criteria were included in the review: (a) The study population consisted of typical developing preschool children, aged 2 to 7 years at the baseline (or the mean or median ages of the study population ranging between 3.0 and 6.0 years). (b) The determinant was continuous PA (total PA, light PA, moderate PA, vigorous PA or MVPA) and/or sedentary behaviour, measured objectively with an accelerometer. (c) The outcome was adiposity (eg, percentage of body fat, BMI, weight status, waist circumference, fat mass and skinfold thickness). (d) The studies were observational studies (cross‐sectional, case control and prospective cohort studies) in which an association between PA or sedentary behaviour and adiposity was quantitatively assessed. (e) Papers were full text, peer reviewed, irrespective of language. To ensure maximal inclusion of studies, cross‐sectional studies were also included if the determinant was adiposity and the outcome was PA.

The following criteria were used to exclude studies: (a) the type of publication (eg, study protocols, reviews or critiques were excluded), (b) studies focussing on children with a (chronic) disease or those confined to hospitals and (c) studies in which PA was measured only during a part of the day (eg, during hours of childcare).

### 2.3 Risk of bias assessment

The risk of bias of the included studies was assessed by two reviewers, independently, using the “Quality of Prognosis Studies in Systematic Reviews” tool (QUIPS).^17^ QUIPS comprises six domains covering the following topics: (a) study participation, (b) study attrition, (c) PA measurement (which is considered valid and reliable if PA is measured for at least 10 h day^−1^ over three valid days), (d) measurement of adiposity outcomes, (e) study confounding (the risk of bias was considered high if the analyses had not been adjusted for sex) and (f) analysis and reporting. The extensive operationalization of the items is described in Appendix B.

For all of the identified articles, each domain was rated as entailing a low, moderate‐ or high risk of bias by two independent reviewers (R. W. and B. H.). Disagreements were discussed in a consensus meeting, and in case of persistent disagreement, a third reviewer (E. H.) was consulted. The overall percentage agreement and Cohen's kappa were calculated to assess the level of inter‐rater agreement. Overall, an item was considered to entail a high risk of bias if scores of more than 50% of the total number of participants revealed a moderate‐ or high risk of bias.

### 2.4 Data extraction

The following data were extracted by two independent reviewers (R. W. and B. H.) from each of the studies: the study population characteristics, participants' characteristics, PA assessment method (the type of accelerometer used, accelerometer cut‐off points, epoch length and minimum wear time), adiposity outcome and, if applicable, definitions applied for overweight/obesity, and statistical analyses and results.

### 2.5 Statistical analysis

Separate meta‐analyses were performed using the Comprehensive Meta Analysis (CMA) software for varying PA intensities and adiposity outcome measures. These analyses were performed if at least two studies were identified, except when differences between studies were considered too large. If multiple articles included the same participants (eg, children of the same cohort) and reported on the same adiposity outcome, one article was selected according to the following criteria: (a) the usefulness of the reported estimate for the meta‐analysis, (b) the longest follow‐up duration, (c) the largest sample size and (d) the estimate with the largest possible effect. Estimates extracted from the articles with continuous outcomes were converted to standardized bèta (stdβ) and standard errors (SEs). If multiple estimates were reported, the adjusted estimates were used for the meta‐analyses. For the dichotomous outcome weight status (non‐overweight vs. overweight/obesity), standardized mean differences were calculated and converted into Pearson correlation coefficients. Since our aim was to examine the effects of PA on adiposity, estimates obtained for studies in which adiposity was the determinant and PA was the outcome were transformed using unadjusted estimates, univariate analyses or Pearson correlation coefficients prior to their inclusion in the meta‐analyses.

If information required for calculating the estimates was missing, the authors of the article were contacted and requested to provide this information. Appendix C presents an overview of the extracted data from the studies used for the meta‐analyses.

Random effect meta‐analyses were performed. Heterogeneity was assessed using the χ^2^ test and the *I* ^2^ statistic.^18^ If study results were found to be heterogeneous (*I* ^2^ > 50% and/or χ^2^ test *P*‐value <.05), an overall estimate was not calculated. The following subgroup analyses were performed to explore heterogeneity: (a) sex (boys, girls, adjusted for sex and not adjusted for sex), (b) epoch length (5‐, 10‐, 15‐ and 60‐s epoch length), (c) type of accelerometer (triaxial vs. uniaxial), (d) prevalence of overweight (studies with a low prevalence of overweight/obesity, ie, <20%, compared with studies with a high prevalence of overweight/obesity, ie, >20% overweight) and (e) individual QUIPS items with a moderate‐ or high risk of bias (studies with a low risk of bias for these items compared with studies with a moderate or high risk of bias).

### 2.6 Publication bias

We conducted funnel plots (a visual inspection) and Egger's test to assess the likelihood of publication bias if at least 10 studies were identified. We created separate funnel plots for different PA intensities and adiposity outcomes. We assumed that there was potential publication bias if the *P*‐value of the Egger's test was <.10.^19^

## 3 RESULTS

**Figure 1:**
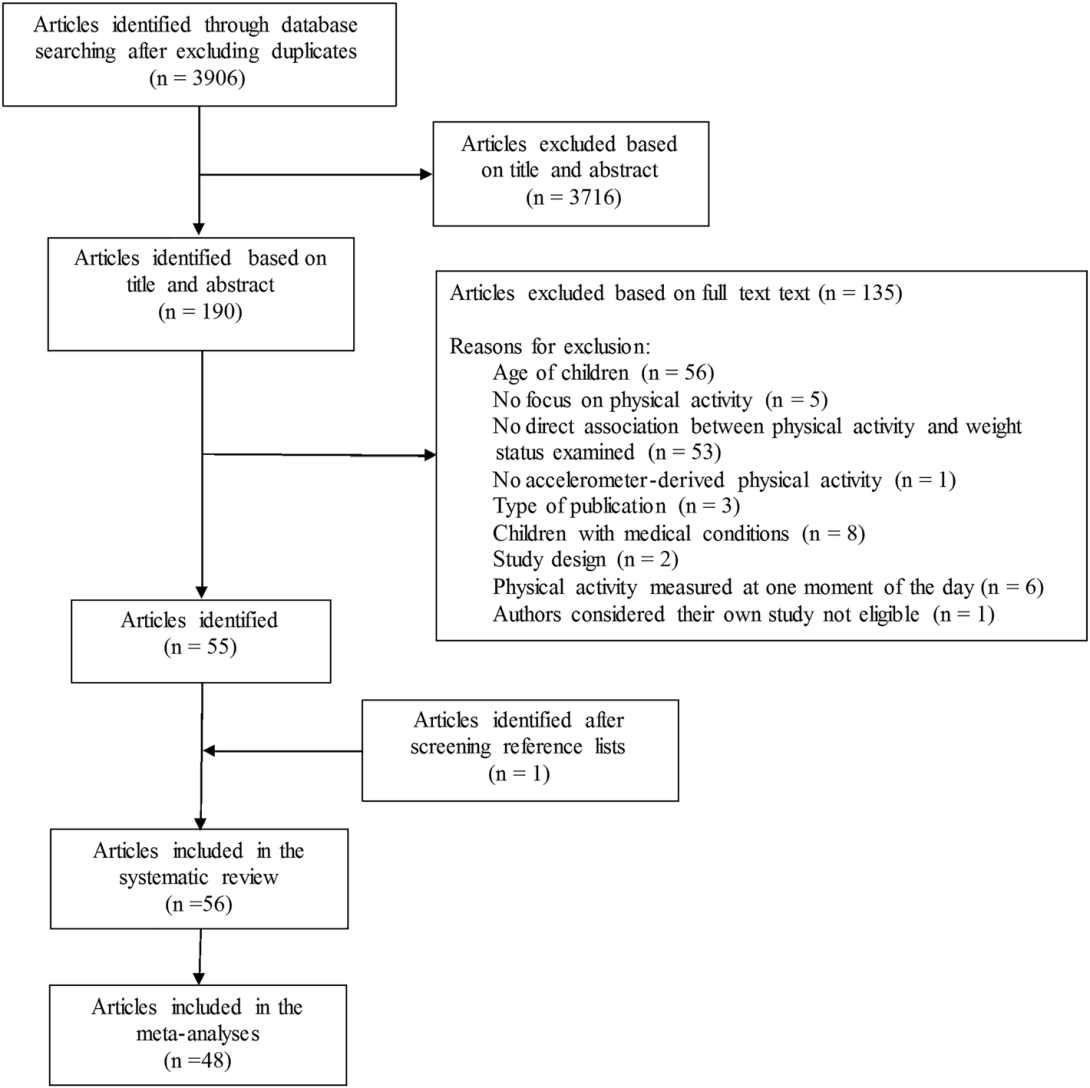
Flow chart of study selection process

We identified 3,906 articles after excluding duplicate articles (2,352). We first read titles and abstracts and subsequently screened the full text of 190 articles, leading to the inclusion of 55 articles. One article was added after we screened the reference lists of these articles. Thus, a total of 56 articles were included in the study, of which 48 were used for the meta‐analyses. Figure 1 shows a flow chart of the process of selecting articles. The individual characteristics of each of the reviewed studies for each adiposity outcome are shown in Appendix D. Most studies were performed in the United States and in Western Europe. With regard to the assessment of PA, a wide variety of methods was observed (Appendix E). Triaxial, biaxial and uniaxial accelerometers were used in 20, 1 and 32 studies, respectively. In two studies, triaxial as well as uniaxial accelerometers were used. Thirty‐two studies used epoch recordings ≤15‐s and 21 studies used 60‐s epoch recordings. With regard to the measurement of adiposity, two different methods to measure percentage of body fat or (trunk) fat mass (index) were observed (dual energy X‐ray absorptiometry and air‐displacement plethysmography), and various locations or numbers of measurements were observed for waist circumference and skinfold thickness (Appendix F). In addition, eight different definitions for overweight and obesity were used (Appendix D).

### 3.1 Risk of bias

Items with a high risk of bias (ie, moderate/high risk of bias in more than 50% of the total number of participants) were (2a) “adequate response rate” (89%), (2c) “no important differences among participants who completed the study with accelerometer data and those who did not” (72%) and (3b) “method of determinant measurement is adequately valid and reliable” (87%) (Appendix G). The overall inter‐rater agreement was 96% (kappa statistic of 0.94), and ranged between 82% for item 2b (“reason for loss of data”) and 100% for several other items.

### 3.2 Association between accelerometer‐derived PA and adiposity

For the association between PA and adiposity, seven longitudinal studies were selected, of which three examined the percentage of body fat, five examined BMI, one examined waist circumference, four examined the fat mass (index) and one examined skinfold thickness.

Additionally, for the cross‐sectional assessment of the association between PA and adiposity, 6 studies were selected on body fat percentage, 23 studies for BMI, 18 studies for weight status, 5 studies for waist circumference, 6 studies for (trunk) fat mass (index) and 3 studies for skinfold thickness.

#### 3.2.1 Outcome: Percentage body fat

##### 3.2.1.1 Longitudinal studies

Three longitudinal studies examined the association between different PA intensities and percentages of body fat. Although the number of longitudinal studies was sufficient, no meta‐analysis was performed because of wide variations among the studies. One study found that children who spent more time engaged in moderate PA, vigorous PA or MVPA had a lower percentage of body fat compared with their peers 12 months later.^20^ Another study did not find an association between total PA, moderate PA or vigorous PA with changes in the percentage of body fat being evident after 9 months.^21^ The third study did not find any relation between total PA, sedentary behaviour or MVPA and 1‐year changes in the percentage of body fat.^22^

##### 3.2.1.2 Cross‐sectional studies

The association between different PA intensities and the percentage of body fat was examined in six cross‐sectional studies.^21^, ^22^, ^23^, ^24^, ^25^, ^26^ The total sample comprised 1,555 children, with 100 to 434 children per study. The prevalence of overweight ranged between 8.5 and 20.1%.

**Figure 2:**
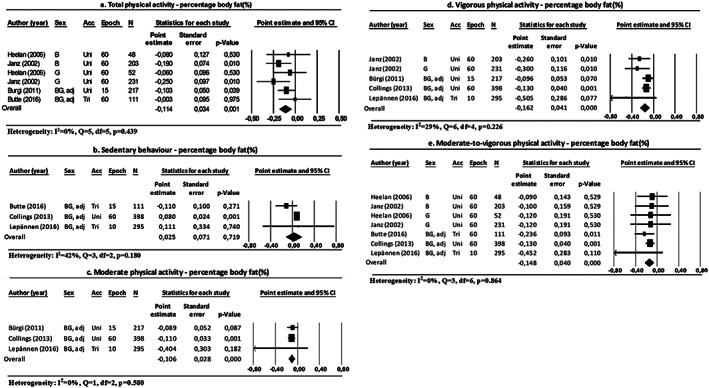
Forest plots of the association between physical activity and body fat percentage, differentiated by physical activity intensities. Abbreviations: B: boys; G: girls; BG: boys and girls; adj: adjusted for sex; acc: accelerometer type; uni: uniaxial; bi: biaxial; tri: triaxial; epoch: epoch length (s); N: number of participants

Pooled estimates indicated that there was an association between total PA, moderate PA, vigorous PA or MVPA and percentage body fat (Figure 2). No association was found between time spent in sedentary behaviour and the percentage of body fat (Figure 2B). Furthermore, one study showed that children who spent more time engaged in light PA had a lower percentage of body fat compared with their peers.^23^ In sum, children who spent more time engaged in total PA, light PA, moderate PA, vigorous PA or MVPA had a lower percentage of body fat compared with their peers.

#### 3.2.2 Outcome: BMI

##### 3.2.2.1 Longitudinal studies

Five longitudinal studies examined the association between different PA intensities and BMI. Although the number of longitudinal studies was sufficient, no meta‐analysis was performed because of wide variations among the studies. One study showed that children who spent more time engaged in MVPA exhibited greater changes in BMI after 1 year compared with their peers.^22^ Another study reported that a 5% increase in total PA or light PA resulted in decreased zBMI in heavier boys 1 year later.^27^ A 5% increase in MVPA resulted in decreased zBMI in normal weight and heavier boys and heavier girls 1 year later.^27^ A third study indicated that children who spent more time engaged in vigorous PA had a higher BMI 12 months later compared with their peers.^20^ The fourth study did not find any differences in BMI gain for low/medium and high MVPA.^28^ The final study did not find any correlations between minutes spent in MVPA and changes in BMI.^29^

##### 3.2.2.2 Cross‐sectional studies

Twenty‐three cross‐sectional studies investigated the association between different PA intensities and BMI.^22^, ^24^, ^26^, ^30^, ^31^, ^32^, ^33^, ^34^, ^35^, ^36^, ^37^, ^38^, ^39^, ^40^, ^41^, ^42^, ^43^, ^44^, ^45^, ^46^, ^47^, ^48^, ^49^ Fifteen studies provided sufficient data to be included in the meta‐analyses,^22^, ^24^, ^26^, ^31^, ^32^, ^33^, ^37^, ^39^, ^40^, ^41^, ^42^, ^43^, ^44^, ^45^, ^49^ and two studies provided additional data on request.^34^, ^35^ Of the two studies that used the same sample,^38^, ^43^ one was chosen according to our predefined criteria for the meta‐analysis.^43^ The authors of three studies informed us that the data were no longer available^30^, ^36^, ^46^ and we failed to contact the authors of two other studies. The total sample comprised 3,502 children, with 46 to 394 children per study. The prevalence of overweight ranged from 8.5 to 28.0%.

**Figure 3:**
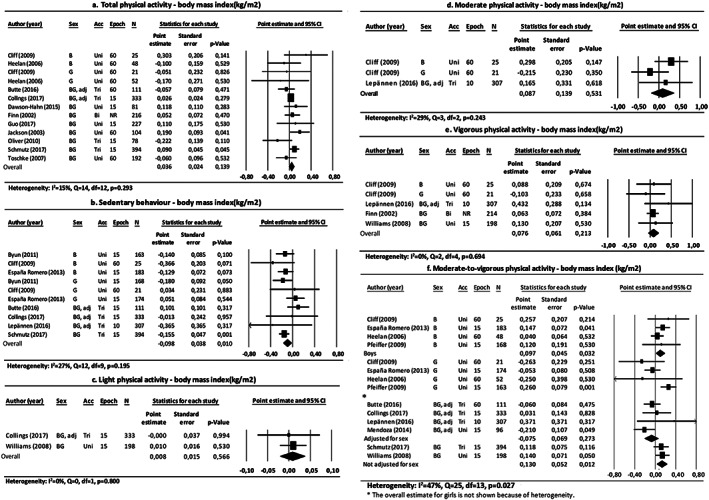
Forest plots of the association between physical activity and body mass index, differentiated by physical activity intensities. Abbreviations: B: boys; G: girls; BG: boys and girls; adj: adjusted for sex; acc: accelerometer type; uni: uniaxial; bi: biaxial; tri: triaxial; epoch: epoch length (s); N: number of participants; NR: not reported

Pooled estimates showed no associations between total PA, light PA, moderate PA or vigorous PA and BMI (Figure 3). By contrast, there was an association between time spent in sedentary behaviour and BMI (Figure 3B). Notably, children who spent less time being sedentary had a higher BMI compared with their peers.

The overall result for the association between MVPA and BMI was heterogeneous (*P* = .027). In subgroup analyses, heterogeneity could be explained by sex, type of accelerometer, epoch length, PA assessment and missing data (Table 1). Pooled estimates in the subgroup analyses for sex showed that boys who spent more time in MVPA had a higher BMI compared with their peers. However, no association was observed for boys and girls together (Figure 3F).

**Figure 4:**
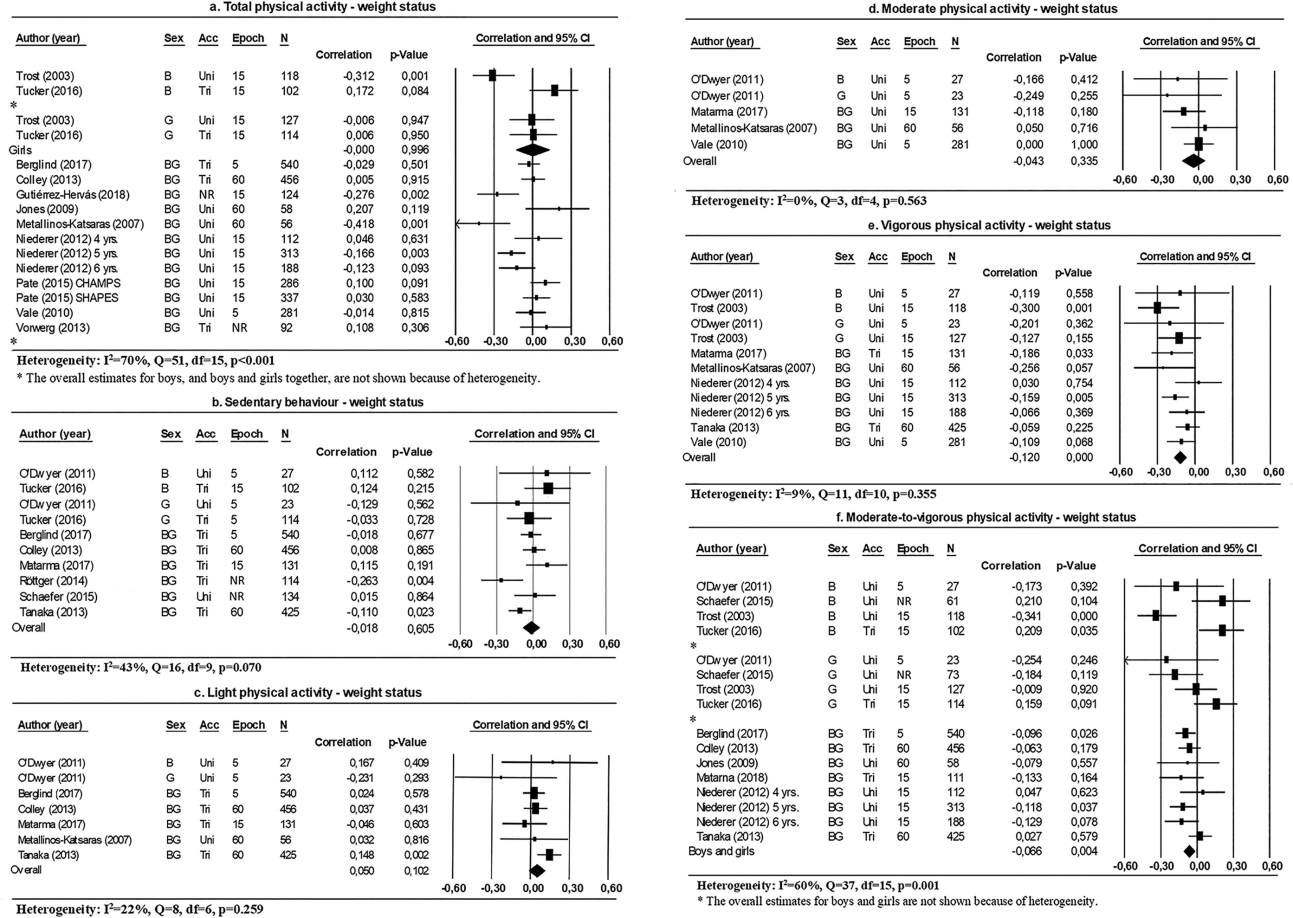
Forest plots of the association between physical activity and weight status, differentiated by physical activity intensities. Abbreviations: B: boys; G: girls; BG: boys and girls: acc: accelerometer type; uni: uniaxial; bi: biaxial; tri: triaxial; epoch: epoch length (s); N: number of participants; NR: not reported

#### 3.2.3 Outcome: Weight status

##### 3.2.3.1 Cross‐sectional studies

Nineteen cross‐sectional studies reported on the association between different PA intensities and weight status (non‐overweight vs. overweight/obesity).^51^, ^52^, ^53^, ^54^, ^55^, ^56^, ^57^, ^58^, ^59^, ^60^, ^61^, ^62^, ^63^, ^64^, ^65^, ^66^, ^67^, ^68^, ^69^ Sixteen studies provided sufficient data and were therefore included in the meta‐analyses,^51^, ^52^, ^54^, ^55^, ^56^, ^57^, ^58^, ^59^, ^60^, ^61^, ^62^, ^64^, ^65^, ^66^, ^67^, ^69^ and for one study, we requested and obtained additional data.^63^ We failed to contact the authors of one study. The same sample was used in two studies,^53^, ^59^ one of which was chosen for the meta‐analysis.^59^ The total sample comprised 4,327 children, with 50 to 540 children per study. The overweight percentage ranged from 7.1 to 43.0%.

Pooled estimates showed that children with overweight spent less time engaged in vigorous PA compared with children who were not overweight (Figure 4E). Time spent in sedentary behaviour, light PA and moderate PA were not associated with weight status (Figure 4).

**Table 1:** The results of the subgroup analyses for body mass index and weight status^a^

Body Mass Index		*Q*	df	*P*‐value(*Q*)	*I* ^2^, %	N	Stdβ ± SE	*P*‐value
Moderate‐to‐vigorous physical activity								
Sex	Boys	2	3	.596	0	4	0.097 ± 0.045	.032
	Girls	11	3	.012	72	‐	‐	‐
	Adjusted for sex	4	3	.305	17	4	‐0.075 ± 0.069	.273
	Not adjusted for sex	0	1	.832	0	2	0.130 ± 0.052	.012
Accelerometer	Triaxial	3	3	.349	9	4	0.044 ± 0.056	.432
	Uniaxial	21	9	.013	57	‐	‐	‐
Epoch length	10s	‐	‐	‐	‐	1	0.371 ± 0.371	.317
	15s	17	7	.016	59	‐	‐	‐
	60s	4	4	.385	4	5	0.000 ± 0.051	.998
Physical activity assessment^b^	Low risk	3	2	.194	39	3	0.049 ± 0.081	.548
	Moderate/high risk	21	10	.021	53	‐	‐	‐
Missing data^b^	Low risk	5	4	.253	25	5	0.032 ± 0.062	.607
	Moderate/high risk	19	8	.017	57	‐	‐	‐

Weight Status		*Q*	df	*P*‐value(*Q*)	*I* ^2^, %	N	*r*	*P*‐value
Total physical activity								
Sex	Boys	13	1	.000	92	‐	‐	‐
	Girls	0	1	.927	0	12	‐0.000	.996
	Boys and girls	37	11	.000	70	‐	‐	‐
Accelerometer	Triaxial	4	4	.361	8	5	0.014	.641
	Uniaxial	36	9	0	75	‐	‐	‐
	Unknown	‐	‐	‐	‐	1	‐0.276	.002
Prevalence of overweight^c^	Low prevalence	9	4	.074	53	‐	‐	‐
	High prevalence	42	10	<.001	76	‐	‐	‐
Physical activity assessment^b^	Low risk	6	1	.011	84	‐	‐	‐
	Moderate/high risk	43	13	<.001	70	‐	‐	‐
Missing data^b^	Low risk	3	2	.207	37	3	0.025	.503
	Moderate/high risk	43	12	<.001	72	‐	‐	‐
Response rate^b^	Low risk	4	2	.158	56	‐	‐	‐
	Moderate/high risk	42	12	<.001	71	‐	‐	‐
Moderate‐to‐vigorous physical activity								
Sex	Boys	22	3	.000	86	‐	‐	‐
	Girls	7	3	.085	55	‐	‐	‐
	Boys and girls	8	7	.364	9	8	‐0.066	.004
Accelerometer	Triaxial	15	5	.010	67	‐	‐	‐
	Uniaxial	18	9	.035	50	‐	‐	‐
Prevalence of overweight^c^	Low prevalence	5	4	.344	11	5	‐0.056	.041
	High prevalence	33	10	<.001	70	‐	‐	‐
Physical activity assessment^b^	Low risk of bias	‐	‐	‐	‐	1	‐0.096	.026
	Moderate/high risk	36	14	.001	61	‐	‐	‐
Missing data^b^	Low risk	‐	‐	‐	‐	1	‐0.096	.026
	Moderate/high risk	36	14	.001	61	‐	‐	‐
Response rate^b^	Low risk	3	2	.274	23	3	‐0.087	.066
	Moderate/high risk	34	12	.001	64	‐	‐	‐

Abbreviation: QUIPS: Quality of Prognosis Studies in Systematic Reviews.

a The results of the subgroup analyses are only shown if results were homogeneous.

b Studies with low risk of bias on this QUIPS item compared with studies with a moderate/high risk of bias.

c High prevalence of overweight was defined as >20% of the study sample affected by overweight or obesity.

The overall results for the association between total PA or MVPA and weight status were heterogeneous (*I* ^2^ = 70% and 60%, respectively). Sex, accelerometer types, missing data, response rates and the prevalence of overweight all explained heterogeneity within subgroup analyses (Table 1). The pooled estimates in the subgroup analyses for sex showed a negative association between MVPA and the weight status of boys and girls considered together (Figure 4F). Moreover, pooled estimates showed no association between total PA and the weight status for girls (Figure 4A).

#### 3.2.4 Outcome: Waist circumference

##### 3.2.4.1 Longitudinal study

One longitudinal study examined the association between MVPA and waist circumference. No relation was found between minutes in MVPA and 1‐ to 3‐year changes in waist circumference.^29^

##### 3.2.4.2 Cross‐sectional studies

Five cross‐sectional studies that examined the association between different PA intensities and waist circumference were identified.^26^, ^33^, ^41^, ^49^, ^70^ The total sample comprised 1,198 children, with 78 to 357 children per study. The prevalence of overweight or a high waist circumference ranged from 8.5 to 58.0%.

Pooled estimates showed that total PA and time spent in sedentary behaviour were not associated with waist circumference (Appendix H). Single studies on the association of light PA,^33^ moderate PA^26^ or vigorous PA,^26^ respectively, with waist circumference did not indicate an association.

The results for the association between MVPA and waist circumference were heterogeneous (*I* ^2^ = 51%). Epoch length, overweight prevalence and PA assessment were factors explaining heterogeneity within subgroup analyses (Appendix I).

#### 3.2.5 Outcome: Fat mass

##### 3.2.5.1 Longitudinal studies

The association between different PA intensities and fat mass was examined in three longitudinal studies. Although the number of longitudinal studies was sufficient, no meta‐analyses were performed because of wide variations among the studies. One study showed that girls with a high baseline MVPA gained less fat mass at the age of 8 years compared with their peers, but no relation was found for boys.^28^ In a second study, boys aged 5 years, with high MVPA levels, showed a lower fat mass at the age of 8 years compared with their peers.^71^ However, no such relation was found for girls. No relations between total PA, sedentary behaviour or MVPA and 1‐year changes in fat mass were found in the last study.^22^

##### 3.2.5.2 Cross‐sectional studies

Four cross‐sectional studies focused on the association between different PA intensities and fat mass.^22^, ^24^, ^25^, ^72^ The total sample comprised 436 children, with 100 to 1,081 children per study. Only one study reported on the prevalence (18%) of overweight.^22^

Pooled estimates showed that children who spent more time in total PA or MVPA had a lower fat mass compared with their peers (Appendix H). One study each examined the association of sedentary behaviour,^22^ light PA^72^ or vigorous PA^25^ with fat mass. Children who spent more time engaged in vigorous PA had a lower fat mass compared with their peers.^25^ No associations were found for sedentary behaviour or light PA with fat mass.^22^, ^72^ In addition, no study focused on moderate PA.

#### 3.2.6 Outcome: Fat mass index

##### 3.2.6.1 Longitudinal studies

One longitudinal study examined the association between different PA intensities and the fat mass index. The results showed no relations existing between sedentary behaviour, moderate PA, vigorous PA or MVPA and fat mass index.^20^

##### 3.2.6.2 Cross‐sectional studies

Two cross‐sectional studies reported on the association between different PA intensities and the fat mass index (fat mass adjusted for height, kg m^−2^).^23^, ^26^ The total sample comprised 693 children (295 children in one study and 398 children in the other). The prevalence of overweight in these studies was 8.5% and 20.1%, respectively.

Pooled estimates showed that children who spent less time engaged in sedentary behaviour or more time in moderate PA, vigorous PA or MVPA had a lower fat mass index compared with their peers (Appendix H). In addition, one study, which examined the association between light PA and fat mass index, did not find an association.^23^ Furthermore, no study focused on total PA.

#### 3.2.7 Outcome: Trunk fat mass (index)

##### 3.2.7.1 Cross‐sectional studies

Only one cross‐sectional study examined the association between different PA intensities and trunk fat mass.^25^ Children who spent more time engaged in vigorous PA had a lower trunk fat mass compared with their peers. A similar association for total PA was only found for girls. No association was found between MVPA and trunk fat mass.^25^

Additionally, the association between different PA intensities and trunk fat mass index (trunk fat mass adjusted for height, kg m^−2^) was examined in one cross‐sectional study.^23^ Children who spent less time engaged in sedentary behaviour or more time in moderate PA, vigorous PA or MVPA had a lower trunk fat mass index compared with their peers. No association was observed between light PA and trunk fat mass index.^23^

#### 3.2.8 Outcome: Skinfold thickness

##### 3.2.8.1 Longitudinal study

The findings of one longitudinal study in which the association between MVPA and skinfold thickness was examined did not reveal any relation between minutes spent in MVPA and changes in skinfold thickness.^29^

##### 3.2.8.2 Cross‐sectional studies

Three cross‐sectional studies examined the association between different PA intensities and skinfold thickness.^33^, ^38^, ^50^ The total sample comprised 811 children, with 309 to 346 children per study. Only one study reported on the prevalence (19.5%) of overweight.^33^

Pooled estimates showed that children who spent more time engaged in total PA, light PA or MVPA had a lower skinfold thickness compared with their peers (Appendix H). One study, in which the association between sedentary behaviour and skinfold thickness was examined, showed no association between time spent in sedentary behaviour and skinfold thickness.^33^ In addition, no studies focused on moderate PA or vigorous PA.

##### 3.2.8.3 Other adiposity outcomes

Two cross‐sectional studies focused on other adiposity outcomes for which no meta‐analyses were conducted. One study focused on central obesity and showed that boys with central obesity spent more time engaged in sedentary behaviour compared with boys who were not affected by central obesity. No differences were found for girls.^73^ The second study focused on stunted‐overweight, revealing differences in sedentary behaviour, light PA and MVPA between stunted children without overweight, stunted children with overweight, non‐stunted children with overweight and non‐stunted children without overweight^74^ Children with overweight spent more time engaged in total PA and light PA compared with stunted children without overweight and less time engaged in sedentary behaviour compared with stunted children with and without overweight. Furthermore, stunted children with and without overweight spent less time engaged in MVPA compared with non‐stunted children without overweight.^74^

#### 3.2.9 Publication bias

No indications of publication bias relating to the associations between different PA intensities and adiposity outcomes were found (all *P* >.10).

## 4 DISCUSSION

We examined the association between accelerometer‐derived PA and varying adiposity outcomes in preschool children using outcome data from 56 studies. Our meta‐analyses showed that the associations between PA and adiposity in preschool children are highly dependent on the intensity of PA and the type of outcome used for assessing the degree of adiposity. There was substantial evidence of an association between (moderate‐to‐) vigorous PA and adiposity.

### 4.1 PA intensity

Children who spent more time engaged in vigorous PA or MVPA showed lower levels of adiposity, revealing that high PA intensities are indicative of positive associations between PA and adiposity at young ages. These findings are in accordance with the results of a systematic review in which the relation between PA and health indicators in children aged 5‐17 years was explored.^75^ The most persistent associations between PA and health indicators were found for higher PA intensities.^75^ We found that associations between PA and adiposity were highly dependent on the PA intensity. This finding highlights the importance of distinguishing between different PA intensities. For interventions among preschool children, we recommend, in accordance with the 24‐h movement guidelines from Canada and Australia,^76^, ^77^ focusing on increasing (moderate‐to‐) vigorous PA to address the growing prevalence of overweight and obesity in young children.

The literature on light PA is scarce compared with vigorous PA and MVPA. The Advisory Committee of the 2018 US PA Guidelines decided to include light PA in their recommendations until more evidence on light PA becomes available.^78^ In our study, meta‐analyses for the association between light PA and percentage of body fat, fat mass or waist circumference were not conducted because of insufficient numbers of studies. However, an association was found between light PA and skinfold thickness, and a trend was observed for weight status. Children spent time of the day entailing the most activity engaged in light PA, which is less intensive than MVPA. It may be easier within intervention studies to increase the time spent in light PA than that spent in high intensity PA. Therefore, future studies should comprehensively examine the influence of light PA on adiposity in preschool children.

### 4.2 Sedentary behaviour

The studies included in this review showed no clear association between sedentary behaviour and adiposity. Associations were found between sedentary behaviour and BMI or fat mass index, but these results should be treated with caution. First, the results showed that children who were less sedentary had a higher BMI compared with their peers. This can probably be explained by the fact that BMI may include not only fat mass but also fat‐free mass or muscle mass. In addition, as children mature, they gain height and their motor‐ability develops as well, which may have influenced the observed association as well. Second, only two studies examining the fat mass index were found. Of these studies, one study reported a positive association between sedentary behaviour and fat mass index, whereas the other study found no association. In addition, a review of studies on children and adolescents conducted in 2012 did not find any associations between accelerometer‐derived sedentary behaviour and adiposity.^9^ Significant negative associations of sedentary behaviour and weight status only occurred in studies in which the former was self‐reported by children and parents. This self‐reported sedentary behaviour was based on reports of children's screen‐time. However, screen‐time and sedentary behaviour are not identical. On the one hand, screen‐time is defined as the time spent engaged in screen‐based behaviours, which can be performed when individuals are sedentary as well as physically active.^79^ On the other hand, sedentary behaviour is defined as sitting, reclining or lying down and entails low energy costs (<1.5 METs).^79^, ^80^ Screen‐time in children is related to a lower vegetable and fruit intake and higher snack consumption.^81^, ^82^ Therefore, an alternative explanation for associations between screen‐time and adiposity may be an excess of energy intake rather than a lack of energy expenditure. Consequently, future studies need to clearly distinguish between screen‐time and sedentary behaviour. Although screen‐time behaviour could be (indirectly) related to adiposity, we found no association between accelerometer‐derived sedentary behaviour and adiposity at young ages.

### 4.3 Adiposity outcome

The findings of this review show that the association between PA and adiposity is highly dependent on the outcome measure used for adiposity. Children who spend more time engaged in PA show a lower percentage of body fat, less fat mass and a lower weight status compared with their peers. However, more time spent in PA is not associated with a lower BMI or waist circumference regardless of PA intensity. These findings are in accordance with a review from 2011 that focused on the association between objectively measured PA and adiposity in preschool children.^11^ The review included the percentage of body fat, BMI, weight status and fat mass as outcome measures for adiposity and partly included the same studies that we reviewed.^11^ The results depended on the outcome measure used for adiposity, with more confirmative results for percentage of body fat than for BMI.^11^ Our review and meta‐analyses were more comprehensive as we also incorporated waist circumference, fat mass index, trunk fat mass (index) and skinfold thickness as adiposity outcome measures. Moreover, we included more recent literature: 38 of the studies in our review and meta‐analyses were published between 2011 and 2018. More confirmative results were found for the percentage of body fat, fat mass and weight status.

This leads to the question of which adiposity outcome should be assessed in future studies. BMI and waist circumference calculations are based on children's anthropometry. As previously mentioned, these are not exclusive measures for fat mass. In this case, the higher BMI could indicate a higher muscle mass instead of a higher fat mass. The body fat percentage, fat mass and skinfold thickness are more precise and may be better measures of adiposity. In addition, although no association between vigorous PA and BMI was found, children who spent more time in vigorous PA are likely to have a lower weight status compared with their less active peers. This may seem contradictory, as weight status is calculated based on BMI. BMI is a continuous measure, whereas weight status is a categorical variable. By dividing children into overweight or obesity and non‐overweight groups, the distinctiveness becomes larger. It may mean that vigorous PA affects adiposity in higher ranges, preventing children to reach a threshold, but it may not affect adiposity over the whole range of BMI and all degrees of adiposity. In other words, it does not make lean children leaner. Therefore, future studies should include the percentage of body fat as outcome for adiposity, and, if BMI data are collected, children should be divided into the following categories: underweight, normal weight, overweight and obesity to increase the distinctiveness of the outcome measure.

### 4.4 Assessment of PA

The reviewed studies encompassed a wide variety of methods for processing accelerometer‐derived PA, which made them less comparable. Because accelerometers are now used more frequently, the development of new accelerometers and the methodological literature on accelerometers have increased, resulting in the deployment of different kinds of accelerometer cut points, and new epoch lengths. A total of 37 accelerometer cut points were used within all of the studies included in this review. A review conducted in 2017 showed 14 different cut points for sedentary behaviour and 11 different cut points for PA intensities within studies that all used the same accelerometer (ActiGraph GT3X) among preschool children.^83^ A study conducted in 2016 showed that levels of PA intensities varied significantly for different epoch lengths and for different cut points.^84^ In this study, PA was measured using ActiGraph GT3X+ accelerometers in children aged 7 to 11 years, and the data were processed using various methods (six different epoch lengths and five different accelerometer cut points were used). When the epoch lengths increased, sedentary behaviour, moderate PA and vigorous PA decreased, whereas light PA increased. The use of different accelerometer cut points was shown to cause a difference of 200 min estimated time engaged in sedentary behaviour or light PA and approximately 50 min on MVPA.^84^ Thus, different epoch lengths and cut points have considerable impacts on the estimated levels of PA intensities, which potentially also influence the associations of PA with adiposity. However, in the current study, we were unable to show whether the associations between PA and adiposity were stronger or weaker if a short epoch time was used, because of heterogeneous findings in the subgroup analyses or a lack of sufficient studies using different epoch lengths. The application of one universal method to assess accelerometer‐derived PA is essential to tackle the growing inconsistency reported in the literature.

### 4.5 Strengths and limitations

This review is the first to apply meta‐analyses for assessing the association between PA and adiposity in preschool children. We used a wide age range (2‐7 years) for preschool children. In general, the preschool period is described as 2 to 5 years of age. Nevertheless, in many countries children attend preschool until they are 7 years old. It is about the period that children receive structured educational instructions, but in the context of play, when they still have ample opportunity to move around freely. Furthermore, most studies in school‐aged children start around the age of 7, leaving a gap between 5 and 7 years. Additionally, this is the first study that seeks to distinguish between different PA intensities and adiposity outcomes. Consequently, a more detailed picture of the association between PA and adiposity in preschool children emerges. Furthermore, only accelerometer‐derived PA was included, making all of the included studies more comparable, even though differences in the assessment of PA still prompted variety.

A limitation of the present study is that 53 out of the 56 included studies were from high‐income countries. Although this made the results from the included studies more comparable, it would be interesting to examine the association between PA and adiposity in more low‐ and middle‐income countries as well, as part of the double burden of poor lifestyle, where low birth weight and poor growth often exist alongside a transition to more sedentary lifestyles and westernized diets. In addition, several methodological limitations should be noted. Firstly, there was a high risk of bias for most studies. About 63% of the studies had a high risk of bias on approximately 70% of the QUIPS items. Especially, the QUIPS‐item regarding validity and reliability of the measurement of PA scored high risk of bias. In 75% of the studies, PA was not measured according to the advised weartime (≥10 h per day) or the minimum advised number of wearing days (≥3 days). Secondly, although we did not detect publication bias in our study, it is likely that some pooled estimates may be overestimated. Among observational studies, it is expected that studies with positive and strong associations are more likely to be published.^85^ We were only able to assess this likelihood of publication bias in 7 out of the 29 performed meta‐analyses. For the other meta‐analyses, the Egger's test would be underpowered to detect publication bias. Lastly, meta‐analyses were only performed on cross‐sectional studies. Unfortunately, we were not able to conduct meta‐analyses for the longitudinal studies because of a lack of comparable studies. Therefore, the pooled estimates might be overestimated and should be handled with caution. More longitudinal studies with multiple measurements taken for both PA and adiposity in young children are needed. Nevertheless, the current study has yielded detailed insights on the association between daily life PA behaviours and adiposity in young children.

## 5 CONCLUSION

More time spent in (moderate‐to‐) vigorous PA was found to be associated with a lower percentage of body fat, lower weight status, less fat mass, a lower fat mass index and lower skinfold thickness in young children. PA was not associated with BMI or waist circumference, irrespective of PA intensity. Furthermore, sedentary behaviour does not appear to be associated with adiposity, irrespective of adiposity outcomes. In addition, light PA should be examined more extensively, and more longitudinal studies are required using multiple measurements for both PA and adiposity. Moreover, universal guidelines are needed to tackle growing inconsistencies regarding the different methods reported in the literature for assessing PA. We recommend that researchers and policymakers focus on high‐intensity PA behaviours to prevent increases in childhood overweight and obesity, paying particular attention to body fat percentage or weight status as adiposity outcome measures.

## CONFLICT OF INTEREST

None declared.

## Supporting information

Data S1. Supporting Information
Table S1. Sample characteristics, statistical analyses, and the results of all of the reviewed studies, differentiated by adiposity outcomes
