# Supplementary material for: Unravelling the association between accelerometer‐derived physical activity and adiposity among preschool children: A systematic review and meta‐analyses
Source: Obes Rev. 2019 Dec 13;21(2):e12936. doi: 10.1111/obr.12936 (PMC7050502; doi:10.1111/obr.12936)
Supplement: Supplementary file 1 — Data S1. Supporting Information Table S1. Sample characteristics, statistical analyses, and the results of all of the reviewed studies, differentiated by adiposity outcomes [file OBR-21-e12936-s001.pdf]

Appendix A. Search strategies used in EMBASE, MEDLINE, and Web of Science.

MEDLINE:

("Child, Preschool"[Mesh] OR child*[tiab] OR pediatr*[tiab] OR paediatr*[tiab] OR preschool[tiab])

AND

("Body Mass Index"[Mesh] OR "Body Fat Distribution"[Mesh] OR "Waist Circumference"[Mesh] OR "Overweight"[Mesh] OR BMI[tiab] OR body mass index[tiab] OR overweight[tiab] OR obes*[tiab] OR weight status[tiab] OR body composition[tiab] OR body fat[tiab])

AND

("Exercise"[Mesh:NoExp] OR physical activ*[tiab] OR physically activ*[tiab] OR activity level*[tiab] OR exercis*[tiab] OR sedenta*[tiab])
AND
("Accelerometry"[Mesh] OR "Monitoring, Physiologic"[Mesh:NoExp] OR "Actigraphy"[Mesh] OR accelerom*[tiab] OR monitor*[tiab] OR actigraph[tiab])

NOT

(adolescen*[Title] OR adult*[Title])

EMBASE:
('preschool child'/exp OR (child* OR pediatr* OR paediatr* OR preschool):ab,ti)

AND

('body mass'/exp OR 'body fat distribution'/exp OR 'waist circumference'/exp OR 'childhood obesity'/exp OR 'obesity'/de OR (BMI OR ‘body mass index’ OR overweight OR obes* OR ‘weight status*’ OR ‘body composition’ OR ‘body fat’):ab,ti)

AND

('physical activity, capacity and performance'/de OR 'exercise'/de OR (physical activ* OR physically activ* OR activity level* OR exercis* OR sedenta*): ab,ti)

AND

('accelerometry'/exp OR 'physiologic monitoring'/de OR 'actimetry'/exp OR (accelerom* OR monitor* OR actigraph):ab,ti)

AND

('article'/it OR 'conference paper'/it OR 'review'/it)

NOT

((adolescen* OR adult*):ti)

Web Of Science

TS = (child* OR pediatr* OR paediatr* OR preschool)

AND

TS = (BMI OR “body mass index” OR overweight OR obes* OR “weight status” OR “body composition” OR “body fat”)

AND

TS = (“physical activ*” OR “physically activ*” OR “activity level*” OR exercis*)
AND
TS = (accelerom* OR monitor* OR actigraph)

Appendix B. QUIPS-based criteria applied in the assessment of the risk of bias.

The ‘Quality of prognosis Studies in Systematic Reviews’ (QUIPS 2013). QUIPS consists of six domain including the following topics: 1) participation, 2) attrition, 3) determinant measurement, 4) outcome measurement, 5) confounding measurement and account and 6) analysis and reporting. The operationalization of this described in the table below. For all identified articles, each domain will be rated low-, moderate- or high risk of bias by two independent reviewers (RW, BH). Disagreement will be discussed in a consensus meeting or by consulting a third reviewer (EH). The overall percentage agreement and Cohen’s kappa will be calculated.

1. Participation
    a. Description of baseline study sample (at least: age, percentage boys, measure for adiposity)
    b. Adequate description participant recruitment (how were participants recruited; e.g. well baby clinics, childcare centre or flyers. For which purpose; i.e. if recruited for intervention purposes, then 0)
    c. Description of period and place of recruitment
    d. Adequate description of inclusion and exclusion criteria

2. Attrition
    a. Adequate response rate (Response rate > 80%)
    b. Reasons for loss to follow-up or loss of (accelerometer) data are provided
    c. No important differences between participants who completed the study/with accelerometer data and those who did not.

3. Determinant measurement
    a. Clear definition or description of the determinant provided
    b. Method of measurement is adequately valid and reliable (at least 3 valid days of 10h /day of PA measurement)

4. Outcome measurement
    a. Clear definition of the outcome is provided
    b. Method of outcome measurement used is adequately valid and reliable

5. Confounding measurement and account
    a. Important confounders are accounted for in the analysis (at least: sex | preferably: SES and nutrition)
    b.
6. Analysis and reporting
    a. Sufficient presentation of data
    b. Selected statistical models is adequate for the design of the study
    c. No selective reporting of the results

	**Quips domain**	Subdomains
1	Participation	a. Description of baseline study sample (at least: age, percentage boys, measure for adiposity)
		b. Adequate description participant recruitment (how were participants recruited; e.g. well baby clinics, childcare centre or flyers. For which purpose; i.e. if recruited for intervention purposes, then 0)
		c. Description of period and place of recruitment
		d. Adequate description of inclusion and exclusion criteria
2	Attrition	a. Adequate response rate (Response rate > 80%)
		b. Reasons for loss to follow-up or loss of (accelerometer) data are provided
		c. No important differences between participants who completed the study/with accelerometer data and those who did not.
3	Determinant measurement	a. Clear definition or description of the determinant provided
		b. Method of measurement is adequately valid and reliable (at least 3 valid days of 10h /day of PA measurement)
4	Outcome measurement	a. Clear definition of the outcome is provided
		b. Method of outcome measurement used is adequately valid and reliable
5	Confounding measurement and account	a. Important confounders are accounted for in the analysis (at least: sex | preferably: SES and nutrition)
6	Analysis and reporting	a. Sufficient presentation of data
		b. Selected statistical models is adequate for the design of the study
		c. No selective reporting of the results

Appendix C. Overview of the extracted data used for the meta-analyses**.**

Author (year)	Total physical activity	Sedentary behaviour	Light physical activity	Moderate physical activity	Vigorous physical activity	Moderate-to-vigorous physical activity
1) Percentage body fat						
Bürgi et al. (2011)[20]	β (95% CI): -0.003 (-0.007 ; -0.0001), SD PA : 164.0, SD out : 4.8; stdβ ± SE: -0.103 ± 0.050			β (95% CI): -0.006 (-0.014; 0.001) , SD PA: 71.0, SD out : 4.8; stdβ ± SE: -0.089 ± 0.052	β (95% CI): -0.010 (-0.022; 0.001), SD PA: 46.0, SD out : 4.8; stdβ ± SE: -0.096 ± 0.053	
Butte et al. (2016)[22]	β ± SE: -0.001 ± 0.028, SD PA : 22.7, SD out : 6.7; stdβ ± SE: -0.003 ± 0.095	β ± SE: -0.011 ± 0.010, SD PA : 67, SD out : 6.7; stdβ ± SE: -0.110 ± 0.100				β ± SE: -0.066 ± 0.026, SD PA: 24.0, SD out : 6.7; stdβ ± SE: -0.236 ± 0.093
Collings et al. (2013)[25]		r: 0.08, p<0.001; stdβ ± SE: 0.08 ± 0.127	r:-0.05, p<0.05; stdβ ± SE: -0.05 ± 0.023	r:-0.11, p<0.001; stdβ ± SE: -0.11 ± 0.175	r:-0.13, p<0.001; stdβ ± SE: -0.13 ± 0.207	r:-0.13, p<0.001; stdβ ± SE: -0.13 ± 0.207
Heelan and Eisenmann (2006)[26]	Boys, r:-0.08, p>0.05; stdβ ± SE: -0.08 ± 0.127<br><br>Girls, r:-0.06, p>0.05; stdβ ± SE: -0.06 ± 0.096					Boys, r:-0.09, p>0.05; stdβ ± SE: -0.09 ± 0.143<br><br>Girls, r:-0.12, p>0.05; stdβ ± SE: -0.12 ± 0.191
Janz et al. (2002)[27]	Boys, r:-0.19, p<0.01; stdβ ± SE: -0.19 ± 0.074<br><br>Girls, r:-0.25, p:<0.01; stdβ ± SE: -0.25 ± 0.097				Boys, r:-0.26, p<0.01; stdβ ± SE: -0.26 ± 0.101<br><br>Girls, r:-0.30, p<0.01; stdβ ± SE: -0.30 ± 0.116	Boys, r:-0.10, p>0.05; stdβ ± SE: -0.10 ± 0.159<br><br>Girls, r:-0.12, p>0.05; stdβ ± SE: -0.12 ± 0.191

Author (year)	Total physical activity	Sedentary behaviour	Light physical activity	Moderate physical activity	Vigorous physical activity	Moderate-to-vigorous physical activity
Leppänen et al. (2016)[28]		β (95% CI): 0.01 (-0.06; 0.07), SD PA: 49.6, SD out : 4.5; stdβ ± SE: 0.111 ± 0.334		β (95% CI): -0.08 (-0.20; 0.04), SD PA: 22.5, SD out : 4.5; stdβ ± SE: -0.404 ± 0.303	β (95% CI): -0.46 (-0.98; 0.06), SD PA: 4.9, SD out : 4.5; stdβ ± SE: -0.505 ± 0.286	β (95% CI): -0.08 (-0.19; 0.02), SD PA: 25.2, SD out : 4.5; stdβ ± SE: -0.452 ± 0.283
2) Body mass index						
Buck et al. (2015)[33]						Data no longer available
Butte et al. (2016)[22]	β ± SE: -0.005 ± 0.007, SD PA : 22.7, SD out : 2.0; stdβ ± SE: -0.057 ± 0.079	β ± SE: 0.003 ± 0.003, SD PA : 67, SD out : 2.0; stdβ ± SE: -0.101 ± 0.101				β ± SE: -0.005 ± 0.007, SD PA: 24.0, SD out : 2.0; stdβ ± SE: -0.060 ± 0.084
Byun et al. (2011)[34] zBMI		Boys, r:-0.14, p< 0.1; stdβ ± SE: -0.14 ± 0.085<br><br>Girls, r:-0.18, p< 0.05; stdβ ± SE: -0.18 ± 0.092				
Cliff et al. (2009)[35] zBMI	Boys, r:0.303, p:0.141; stdβ ± SE: -0.303 ± 0.206<br><br>Girls, r:-0.051, p:0.826; stdβ ± SE: -0.051 ± 0.232	Boys, r: -0.366, p:0.072; stdβ ± SE: -0.366 ± 0.203<br><br>Girls, r: 0.034, p:0.883; stdβ ± SE: -0.034 ± 0.231		Boys, r: 0.298, p: 0.147; stdβ ± SE: 0.298 ± 0.205<br><br>Girls, r: -0.215, p:0.350; stdβ ± SE: -0.215 ± 0.230	Boys, r: 0.088, p: 0.674; stdβ ± SE: 0.088 ± 0.209<br><br>Girls, r: -0.103, p:0.658; stdβ ± SE: -0.103 ± 0.233	Boys, r: 0.257, p: 0.215; stdβ ± SE: 0.257 ± 0.207<br><br>Girls, r: -0.263, p: 0.250 stdβ ± SE: -0.263 ± 0.229

Author (year)	Total physical activity	Sedentary behaviour	Light physical activity	Moderate physical activity	Vigorous physical activity	Moderate-to-vigorous physical activity
Collings et al. (2017)[36]	β (95% CI): 0.042 (-0.037; 0.12), SD PA : 320.2, SD out : 1.7; stdβ ± SE: 0.026 ± 0.024	β (95% CI): -0.007 (-0.040; 0.027), SD PA : 64.6, SD out : 1.7; stdβ ± SE: -0.013 ± 0.242	β (95% CI): -0.000 (-0.043; 0.042), SD PA: 54.0, SD out : 1.7; stdβ ± SE: -0.0003 ± 0.037			β (95% CI): 0.045 (-0.040; 0.13), SD PA: 23.5, SD out : 1.7; stdβ ± SE: 0.031 ± 0.143
Dawson-Hahn et al. (2015)[38] zBMI	Stdβ: 0.118, p=0.283* stdβ ± SE : 0.118 ± 0.110					Failed to contact authors.
España-Romero et al. (2013)[39] zBMI		Boys, β ± SE: -0.050 ± 0.028, SD PA : 3.1, SD out : 1.2; stdβ ± SE: -0.129 ± 0.072<br><br>Girls, β ± SE: 0.014 ± 0.023, SD PA : 3.3, SD out : 0.9; stdβ ± SE: 0.051 ± 0.084				Boys, β ± SE: 0.080 ± 0.039, SD PA: 2.2, SD out : 1.2; stdβ ± SE: 0.147 ± 0.072<br><br>Girls, β ± SE: -0.024 ± 0.036, SD PA: 2.0, SD out : 0.9; stdβ ± SE: -0.053 ± 0.080
Finn et al. (2002)[40]	p = 0.4; stdβ ± SE: 0.052 ± 0.072*				p = 0.3; stdβ ± SE: 0.063 ± 0.072*	
Fisher et al. (2005)[41] zBMI	Data no longer available					
Guo et al. (2017)[42] zBMI	r: 0.11, p >0.05; stdβ ± SE: 0.11 ± 0.175					
Heelan and Eisenmann (2006)[26]	Boys, r:-0.10, p>0.05; stdβ ± SE: -0.10 ± 0.159<br><br>Girls, r:-0.17, p>0.05; stdβ ± SE: -0.17 ± 0.271					Boys, r:0.04, p>0.05; stdβ ± SE: 0.04 ± 0.064<br><br>Girls, r:-0.25, p>0.05; stdβ ± SE: -0.25 ± 0.398

Author (year)	Total physical activity	Sedentary behaviour	Light physical activity	Moderate physical activity	Vigorous physical activity	Moderate-to-vigorous physical activity
Herzig et al. (2017)[43]	Same participants as Schmutz et al. (2017). Excluded for meta-analysis based on criteria 3.					Same participants as Schmutz et al. (2017). Excluded for meta-analysis based on criteria 3.
Iivonen et al. (2013)[44]	Failed to contact authors.					Failed to contact authors.
Jackson et al. (2003)[45] zBMI	r: 0.19, p: 0.04 ; stdβ ± SE: 0.19 ± 0.093					
Kelly et al. (2006)[46] zBMI	Failed to contact authors.					
Leppänen et al. (2016)[28]		β (95% CI): -0.01 (-0.03; 0.01), SD PA: 49.6, SD out : 1.4; stdβ ± SE: -0.365 ± 0.365		β (95% CI): 0.01 (-0.02; 0.05), SD PA: 22.5, SD out : 1.4; stdβ ± SE: 0.165 ± 0.331	β (95% CI): 0.12 (-0.03; 0.28), SD PA: 4.9, SD out : 1.4; stdβ ± SE: 0.432 ± 0.288	β (95% CI): 0.02 (-0.02; 0.05), SD PA: 25.2, SD out : 1.4; stdβ ± SE: 0.371 ± 0.371
Mendoza et al. (2014)[47]						stdβ: -0.21, p : 0.049; stdβ ± SE: -0.21 ± 0.107
Oliver et al. (2010)[48]	Coefficient (95%CI): -0.04 (-0.08; 0.01), SD BMI : 1.75, SD PA : 0.316*; stdβ ± SE: -0.222 ± 0.139					

Author (year)	Total physical activity	Sedentary behaviour	Light physical activity	Moderate physical activity	Vigorous physical activity	Moderate-to-vigorous physical activity
Pfeiffer et al. (2009)[49] zBMI						Boys: r: 0.12, p: >0.05; stdβ ± SE: 0.12 ± 0.191<br><br>Girls: r: 0.26, p ≤ 0.001; stdβ ± SE: 0.26 ± 0.079
Schmutz et al. (2017)[50] zBMI	r: 0.090; p=0.045* stdβ ± SE: 0.090 ± 0.045	r: -0.155; p=0.001* stdβ ± SE: -0.155 ± 0.047				r: 0.118; p=0.009* stdβ ± SE: 0.118 ± 0.075
Toschke et al. (2007)[52]	r: -0.06, p: >0.05; stdβ ± SE: -0.06 ± 0.096					
Williams et al. (2008)[53] zBMI		r: -0.09, p: >0.05; stdβ ± SE: -0.09 ± 0.143	r: 0.01, p: >0.05; stdβ ± SE: 0.01 ± 0.016		r: 0.13, p: >0.05; stdβ ± SE: 0.13 ± 0.207	r: 0.14, p: <0.05; stdβ ± SE: 0.14 ± 0.071
Yamamoto et al. (2011)[54]						Data no longer available
3) Weight status (mean ± SD or mean (SE))						
Berglind et al. (2017)[55]	NW: 1452.0 ± 263.9<br>OW: 1437.0 ± 243.8;<br>r: -0.029	NW: 341.1 ± 65.4<br>OW: 338.8 ± 64.0;<br>r: -0.018	NW: 365.2± 46.8<br>OW: 367.5 ± 52.2;<br>r: 0.024			NW: 51.5 ± 21.8<br>OW: 47.4 ± 18.4;<br>r: -0.096
Colley et al. (2013)[56]	NW: 349 (5)<br>OW: 350 (15);<br>r: 0.005	NW: 358 (7)<br>OW: 360 (9);<br>r: 0.008	NW: 281 (4)<br>OW: 287 (11);<br>r: 0.037			NW: 68 (2)<br>OW: 63 (5);<br>r: -0.063
Ebenegger et al. (2012)[57]	Same participants as Niederer et al. (2012). Excluded for meta-analysis based on criteria 3.				Same participants as Niederer et al. (2012). Excluded for meta-analysis based on criteria 3.	Same participants as Niederer et al. (2012). Excluded for meta-analysis based on criteria 3.

Author (year)	Total physical activity	Sedentary behaviour	Light physical activity	Moderate physical activity	Vigorous physical activity	Moderate-to-vigorous physical activity
Jones et al. (2009)[61]	NW: 865.7 ± 226.2<br>OW: 961.1 ± 213.1;<br>r: 0.207					NW: 33.0 ± 25.2<br>OW: 29.0 ± 22.6;<br>r: -0.079
Matarma et al. (2017)[62]		NW: 50.0 ± 5.1 %day<br>OW: 51.2 ± 5.5 %day<br>r: 0.115	NW: 41.7 ± 4.2 %day<br>OW: 41.3 ± 5.0 %day<br>r: -0.046	NW: 6.0 ± 1.8 %day<br>OW: 5.6 ± 0.9 %day<br>r: -0.118	NW: 2.3 ± 1.1 %day<br>OW: 1.9 ± 0.8 %day<br>r: -0.186	
Matarma et al. (2018)[64]		Same participants as Matarna et al. 2017. Excluded for meta-analysis based on criteria 3.				NW: 8.2 ± 2.6 %day<br>OW: 7.5 ± 1.6 %day<br>r: -0.133
Metallinos-Katsaras et al. (2007)[65]	p: 0.31,<br>NW: 768.7, OW: 720.6;<br>r: -0.418		p: 0.84,<br>NW: 412.2, OW: 415.6;<br>r: 0.032	p: 0.72,<br>NW: 241.2, OW: 246.4;<br>r: 0.050	p: 0.06,<br>NW: 26.8, OW: 19.9;<br>r: -0.256	
O'Dwyer et al. (2011)[67]		Boys,<br>Weekday:<br>NW: 751.3 ± 146.7<br>OW: 652.6 ± 168.6,<br>Weekend:<br>NW: 684.0 ± 198.1<br>OW: 863.7 ± 164.4;<br>r: 0.112<br><br>Girls,<br>Weekday:<br>NW: 672.4 ± 117.4<br>OW: 668.0 ± 150.8,<br>Weekend:<br>NW: 757.0 ± 203.0<br>OW: 673.0 ± 200.4;<br>r: -0.129	Boys,<br>Weekday:<br>NW: 48.5 ± 15.1<br>OW: 52.9 ± 11.9,<br>Weekend:<br>NW: 64.4 ± 16.2<br>OW: 72.2 ± 22.1;<br>r: 0.167<br><br>Girls,<br>Weekday:<br>NW: 54.0 ± 16.7<br>OW: 50.7 ± 10.0,<br>Weekend:<br>NW: 67.5 ± 19.7<br>OW: 54.7 ± 16.6;<br>r: -0.231	Boys,<br>Weekday:<br>NW: 32.0 ± 16.9<br>OW: 25.3 ± 5.1 ,<br>Weekend:<br>NW: 23.9 ± 7.6<br>OW: 22.4 ± 6.8;<br>r: -0.166<br><br>Girls,<br>Weekday:<br>NW: 28.3 ± 12.1<br>OW: 25.4 ± 7.0,<br>Weekend:<br>NW: 29.8 ± 21.0<br>OW: 18.6 ± 7.4;<br>r: -0.249	Boys,<br>Weekday:<br>NW: 13.2 ± 4.7<br>OW: 13.3 ± 3.3,<br>Weekend:<br>NW: 14.1 ± 5.5<br>OW: 11.6 ± 6.0;<br>r: -0.119<br><br>Girls,<br>Weekday:<br>NW: 15.0 ± 7.3<br>OW: 12.6 ± 4.4,<br>Weekend:<br>NW: 12.6 ± 6.5<br>OW: 10.3 ± 2.3;<br>r: -0.201	Boys,<br>Weekday:<br>NW: 45.2 ± 20.3<br>OW: 38.6 ± 8.1,<br>Weekend:<br>NW: 38.0 ± 10.4<br>OW: 34.0 ± 11.9;<br>r: -0.173<br><br>Girls,<br>Weekday:<br>NW: 43.3 ± 17.0<br>OW: 38.0 ± 10.5,<br>Weekend:<br>NW: 42.4 ± 26.4<br>OW: 28.9 ± 9.5;<br>r: -0.254

Author (year)	Total physical activity	Sedentary behaviour	Light physical activity	Moderate physical activity	Vigorous physical activity	Moderate-to-vigorous physical activity
Gutiérrez-Hervás et al. (2018)[59]	Underweight: 604 ± 128, normal weight: 627 ± 118, overweight: 563 ± 130,<br>obese: 538 ± 114.<br>r: -0.276					
Niederer et al. (2012)[66]	4 years:<br>NW: 712 ± 139<br>OW: 725 ± 153<br>r: 0.046<br>5 years:<br>NW: 740 ± 181<br>OW: 682 ± 130;<br>r: -0.166<br>6 years:<br>NW: 745 ± 165<br>OW: 704 ± 167;<br>r: -0.123				4 years:<br>NW: 8.9 ± 3.4<br>OW: 9.1 ± 3.1<br>r: 0.030<br>5 years:<br>NW: 9.7 ± 4.1<br>OW: 8.4 ± 3.7;<br>r: -0.159<br>6 years:<br>NW: 10.2 ± 9.1<br>OW: 9.1 ± 3.6;<br>r: -0.066	4 years:<br>NW: 34.6 ± 8.7<br>OW: 35.4 ± 7.9<br>r: 0.047<br>5 years:<br>NW: 35.7 ± 9.5<br>OW: 33.5 ± 8.1;<br>r: -0.118<br>6 years:<br>NW: 36.1 ± 9.5<br>OW: 33.7 ± 8.0;<br>r: -0.129
Pate et al. (2015)[68]	CHAMPS:<br>NW: 14.2 (0.3)<br>Overweight: 14.9 (0.5)<br>Obese: 15.2 (0.6);<br>r: 0.100<br><br>SHAPES:<br>NW: 15.3 (0.4)<br>Overweight: 15.5 (0.5)<br>Obese: 15.8 (0.5);<br>r: 0.030					
Röttger et al. (2014)[69]		t: -2.89, df: 97, p: 0.044; r: -0.243				

Author (year)	Total physical activity	Sedentary behaviour	Light physical activity	Moderate physical activity	Vigorous physical activity	Moderate-to-vigorous physical activity
Schaefer et al. (2015)[71]		p: <0.05,<br>NW: 866.3, OW: 867.9;<br>r: 0.015				Boys*,<br>NW: 83.1 ± 27.3<br>OW: 95.6 ± 31.4;<br>r: 0.210<br><br>Girls*,<br>NW: 72.1 ± 27.4<br>OW: 62.5 ± 22.8;<br>r: -0.184
Tanaka and Tanaka (2013)[72]		Thin: 1206 ± 48<br>NW: 1179 ± 51<br>OW: 1172 ± 44;<br>r: -0.110	Thin: 142 ± 28<br>NW: 159 ± 29<br>OW: 165 ± 24;<br>r: 0.148		Thin: 18 ± 9<br>NW: 22 ± 12<br>OW: 20 ± 10;<br>r: -0.059	Thin: 92 ± 27<br>NW: 102 ± 30<br>OW: 102 ± 33;<br>r: 0.027
Trost et al. (2003)[74]	Boys,<br>NW: 60000 ± 14500<br>OW: 50500 ± 14400;<br>r: -0.312<br><br>Girls,<br>NW: 52100 ± 15700<br>OW: 51900 ± 15800;<br>r: -0.006				Boys,<br>NW: 6.7 ± 2.8<br>OW: 4.9 ± 3.1;<br>r: -0.300<br><br>Girls,<br>NW: 5.6 ± 3.7<br>OW: 4.7 ± 3.0;<br>r: -0.127	Boys,<br>NW: 33.7 ± 8.5<br>OW: 27.2 ± 10.5;<br>r: -0.341<br><br>Girls,<br>NW: 28.5 ± 11.1<br>OW: 28.3 ± 10.8;<br>r: -0.009

Author (year)	Total physical activity	Sedentary behaviour	Light physical activity	Moderate physical activity	Vigorous physical activity	Moderate-to-vigorous physical activity
Tucker et al. (2016)[75]	Boys,<br>NW: 20.3 ± 3.6<br>OW: 21.7 ± 5.0;<br>r: 0.172<br><br>Girls,<br>NW: 18.6 ± 3.3<br>OW: 18.6 ± 3.8;<br>r: 0.006	Boys,<br>NW: 39.5 ± 3.7<br>OW: 38.4 ± 5.2;<br>r: 0.124<br><br>Girls:<br>NW: 41.6 ± 3.5<br>OW: 41.3 ± 3.7;<br>r: -0.033				Boys,<br>NW: 2.6 ± 1.4<br>OW: 3.2 ± 1.3;<br>r: 0.209<br><br>Girls:<br>NW: 2.4 ± 1.3<br>OW: 2.8 ± 1.6;<br>r: 0.159
Vale et al. (2010)[76]	NW: 134 ± 36<br>OW: 133 ± 29;<br>r: -0.014			NW: 58 ± 14<br>OW: 58 ± 13;<br>r: 0.00	NW: 38 ± 14<br>OW: 35 ± 12;<br>r: -0.109	
van Cauwenberghe et al. (2012)[77]		Failed to contact authors.				Failed to contact authors.
Vorwerg et al. (2013)[78]	NW: 4.4 ± 1.8<br>OW: 4.8 ± 2.1;<br>r: 0.108					
4) Waist circumference						
Collings et al. (2017)[36]	β (95% CI): -0.072 (-0.39; 0.24), SD PA : 320.2, SD out : 4.7; stdβ ± SE: -0.145 ± 0.094	β (95% CI): 0.056 (-0.075; 0.19), SD PA : 64.6, SD out : 4.7 ; stdβ ± SE: 0.144 ± 0.017	β (95% CI): -0.056 (-0.22; 0.11), SD PA: 54.0, SD out : 4.7; stdβ ± SE: -0.034 ± 0.010			β (95% CI): -0.14 (-0.46; 0.19), SD PA: 23.5, SD out : 4.7; stdβ ± SE: -0.190 ± 0.033

Author (year)	Total physical activity	Sedentary behaviour	Light physical activity	Moderate physical activity	Vigorous physical activity	Moderate-to-vigorous physical activity
España-Romero et al. (2013)[39]		Boys, β ± SE: -0.152 ± 0.113, SD PA : 3.1, SD out : 4.7; stdβ ± SE: -0.100 ± 0.075<br><br>Girls, β ± SE: 0.154 ± 0.117, SD PA : 3.3, SD out : 4.8; stdβ ± SE: 0.106 ± 0.080				Boys, β ± SE: 0.233 ± 0.160, SD PA: 2.2, SD out : 4.7; stdβ ± SE: 0.109 ± 0.075<br><br>Girls, β ± SE: -0.190 ± 0.187, SD PA: 2.0, SD out: 4.8; stdβ ± SE: -0.079 ± 0.078
Leppänen et al. (2016)[28]		β (95% CI): -0.03 (-0.08; 0.02), SD PA: 49.6, SD out : 3.7; stdβ ± SE: -0.407 ± 0.339		β (95% CI): 0.07 (-0.03; 0.16), SD PA: 22.5, SD out : 3.7; stdβ ± SE: 0.430 ± 0.277	β (95% CI): 0.14 (-0.27; 0.55), SD PA: 4.9, SD out : 3.7; stdβ ± SE: 0.187 ± 0.274	β (95% CI): 0.06 (-0.03; 0.14), SD PA: 25.2, SD out : 3.7; stdβ ± SE: 0.413 ± 0.275
Oliver et al. (2010)[48]	Coefficient (95%CI): -0.01 (-0.03; 0.01), SD waist circumference: 3.95, SD PA : 0.316*; stdβ ± SE: -0.125 ± 0.125					
Oliver et al. (2013)[79]		Failed to contact authors.				Failed to contact authors.
5) Fat mass						
Butte et al. (2016)[22]	β ± SE: -0.008 ± 0.008, SD PA, 22.7, SD out: 2.2; stdβ ± SE: -0.083 ± 0.083	β ± SE: 0.002 ± 0.003, SD PA: 67, SD out : 2.2; stdβ ± SE: 0.061 ± 0.091				β ± SE: -0.015 ± 0.008, SD PA: 24.0, SD out : 2.2; stdβ ± SE: -0.164 ± 0.087

Author (year)	Total physical activity	Sedentary behaviour	Light physical activity	Moderate physical activity	Vigorous physical activity	Moderate-to-vigorous physical activity
Heelan and Eisenmann (2006)[26]	Boys, r:-0.08, p>0.05; stdβ ± SE: -0.08 ± 0.127<br><br>Girls, r:-0.13, p>0.05; stdβ ± SE: -0.13 ± 0.207					Boys, r:-0.08, p>0.05; stdβ ± SE: -0.08 ± 0.127<br><br>Girls, r:-0.22, p>0.05; stdβ ± SE: -0.22 ± 0.350
Jackson et al. (2009)[81]	Failed to contact authors.					
Janz et al. (2002)[27]	Boys, r: -0.15, p: <0.05; stdβ ± SE: -0.15 ± 0.077<br><br>Girls, r: -0.19, p: <0.01; stdβ ± SE: -0.19 ± 0.074				Boys, r: -0.22, p: <0.01; stdβ ± SE: -0.22 ± 0.085<br><br>Girls, r: -0.25, p:<0.01; stdβ ± SE: -0.25 ± 0.097	Boys, r: -0.07, p: >0.05; stdβ ± SE: -0.07 ± 0.111<br><br>Girls, r: -0.06, p: >0.05; stdβ ± SE: -0.06 ± 0.096
6) Fat mass index						
Collings et al. (2013)[25]		r:0.058, p<0.01; stdβ ± SE: 0.058 ± 0.023	r:-0.02, p≥0.05; stdβ ± SE: -0.02 ± 0.010	r: -0.073, p<0.01; stdβ ± SE: -0.073 ± 0.028	r: -0.12, p<0.001; stdβ ± SE: -0.12 ± 0.191	r: -0.10, p<0.001; stdβ ± SE: -0.10 ± 0.159
Leppänen et al. (2016)[28]		β (95% CI): 0.00 (-0.02; 0.01), SD PA: 49.6, SD out : 0.9; stdβ ± SE: 0.000 ± 0.270		β (95% CI): -0.01 (-0.03; 0.02), SD PA: 22.5, SD out : 0.9; stdβ ± SE: -0.245 ± 0.367	β (95% CI): -0.04 (-0.15; 0.07), SD PA: 4.9, SD out : 0.9; stdβ ± SE: -0.213 ± 0.293	β (95% CI): -0.01 (-0.03; 0.01), SD PA: 25.2, SD out : 0.9; stdβ ± SE: -0.274 ± 0.274

7) Trunk fat mass						
Author (year)	Total physical activity	Sedentary behaviour	Light physical activity	Moderate physical activity	Vigorous physical activity	Moderate-to-vigorous physical activity
Janz et al. (2002)[27]	Boys, r: -0.13, p: >0.05; stdβ ± SE: -0.13 ± 0.207<br><br>Girls, r: -0.19, p: <0.01; stdβ ± SE: -0.19 ± 0.074				Boys, r: -0.21, p: <0.01; stdβ ± SE: -0.21 ± 0.082<br><br>Girls, r: -0.26, p: <0.01; stdβ ± SE: -0.26 ± 0.101	Boys, r: -0.05, p: >0.05; stdβ ± SE: -0.05 ± 0.080<br><br>Girls, r: -0.06, p: >0.05; stdβ ± SE: -0.06 ± 0.096
8) Trunk fat mass index						
Collings et al. (2013)[25]		r:0.062, p<0.01; stdβ ± SE: 0.062 ± 0.024	r: -0.033, p≥0.05; stdβ ± SE: -0.033 ± 0.017	r: -0.059, p<0.01; stdβ ± SE: -0.059 ± 0.023	r: -0.10, p<0.001; stdβ ± SE: -0.10 ± 0.159	r: -0.084, p<0.001; stdβ ± SE: -0.084 ± 0.134
9) Skinfold thickness						
Collings et al. (2017)[36]	β (95% CI): -0.064 (-1.46; 0.19), SD PA : 320.2, SD out : 4.5; stdβ ± SE: -0.017 ± 0.037	β (95% CI): 0.21 (-0.12; 0.53), SD PA : 64.6, SD out : 4.5; stdβ ± SE: 0.040 ± 0.016	β (95% CI): -0.059 (-0.47; 0.35), SD PA: 54.0, SD out : 4.5; stdβ ± SE: -0.034 ± 0.006			β (95% CI): -0.76 (-1.43; -0.085), SD PA: 23.5, SD out : 4.5 ; stdβ ± SE: -0.037 ± 0.042
Fang et al. (2017)[83]			Boys: Stdβ: 0.026, p>0.05<br>Stdβ ± SE: 0.026 ± 0.041<br><br>Girls : Stdβ: -0.077, p>0.05<br>Stdβ ± SE: -0.077 ± 0.123			Boys: Stdβ: -0.195, p<0.05<br>Stdβ ± SE: -0.195 ± 0.099<br><br>Girls : Stdβ: -0.041, p>0.05<br>Stdβ ± SE: -0.041 ± 0.065
Herzig et al. (2017)[43]	r: -0.12, p: ≤0.05; stdβ ± SE: -0.12 ± 0.061					r: -0.13, p: ≤0.05; stdβ ± SE: -0.13 ± 0.066

Data presented as original from paper; standardized$\beta \pm$ SE or pearson correlation for meta-analysis. SD out: standard deviation of the outcome (ie: percentage of body fat, body mass index, waist circumference, fat mass or skinfold thickness)

* Data received on request

Appendix D. Sample characteristics, statistical analyses, and the results of all of the reviewed studies, differentiated by adiposity outcomes.

1) PERCENTAGE BODY FAT

Longitudinal studies

Author (year)	Country and cohort	Participant characteristics (sample size; age[a])	Prevalence of overweight[b]	Statistical analysis	Conclusion
Bürgi et al. (2011)[1]	Switzerland Ballabeina Study	n = 217, 104 boys, 113 girls; 4 - 6 years, 5.2(0.6)	10.0% (IOTF)[2]	Mixed linear regression adjusted for age, sex, preschool clusters and baseline outcome parameters.	Total PA, moderate PA or vigorous PA was not associated with change of percentage body fat 9 months later.
Butte et al. (2016)[3]	United States	n = 111, 58 boys, 53 girls; 3 - 5 years, 4.6(0.9)	18.0% (CDC)[4]	Mixed-effects linear models adjusted for age, sex, race/ethnicity, daycare hours, household size, household income, mother's age, BMI and education and awake time.	No relation between total PA, sedentary behaviour or MVPA and percentage body fat 1 year later.
Leppänen et al. (2017)[5]	Sweden MINISTOP trial	n = 138, 73 boys, 65 girls; 4 years, 4.5(0.2) follow-up: 5.6(0.2)	7.2% baseline, 6.5% follow-up	Linear regression, adjusted for child's sex, age at measurement, ActiGraph awake wearing time.	Children who spent more time engaged in moderate PA, vigorous PA or MVPA had a lower percentage of body fat 12 months later compared with their peers. No relations for sedentary behaviour.

Cross-sectional studies

Author (year)	Country and cohort	Participant characteristics (sample size; age[a])	Prevalence of overweight[b]	Statistical analysis	Conclusion
Bürgi et al. (2011)[1]	Switzerland Ballabeina Study	n = 217, 104 boys, 113 girls; 4 - 6 years, 5.2(0.6)	10.0% (IOTF)[2]	Mixed linear regression adjusted for age, sex and preschool clusters.	Children who spent more time engaged in total PA had a lower percentage body fat compared with their peers. No results for moderate PA and vigorous PA.
Butte et al. (2016)[3]	United States	n = 111, 58 boys, 53 girls; 3 - 5 years, 4.6(0.9)	18.0% (CDC)[4]	Mixed-effects linear models adjusted for age, sex, race/ethnicity, daycare hours, household size, household income, mother's age, BMI and education and awake time.	Children who spent more time engaged in MVPA had a lower percentage body fat compared with their peers. No results for total PA and sedentary behaviour.

Author (year)	Country and cohort	Participant characteristics (sample size; age[a])	Prevalence of overweight[b]	Statistical analysis	Conclusion
Collings et al. (2013)[6]	United Kingdom SWS	n = 398, 202 boys, 196 girls; 4 years, 4.1(0.1)	20.1% (Cole et al. 2000)[2]	Bivariate correlations.	Children who spent less time engaged in sedentary behaviour or more time engaged in light PA, moderate PA, vigorous PA or MVPA had a lower percentage of body fat compared with their peers.
Heelan and Eisenmann (2006)[7]	United States	n = 100, 48 boys, 52 girls; 4 - 7 years, 5.8(1.3)	-	Partial correlations controlling for chronological age.	No association between total PA or MVPA and percentage body fat.
Janz et al. (2002)[8]	United States IOWA-Fluoride study, IOWA-Bone Development study	n = 434, 203 boys, 231 girls; 4 - 6 years, 5.3(0.4)	-	Partial correlation coefficients adjusted for age and height.	Children who spent more time engaged in total PA or vigorous PA had a lower percentage body fat compared with their peers. No results for MVPA.
Leppänen et al. (2016)[9]	Sweden MINISTOP trial	n = 295, 166 boys, 129 girls; 4.5(0.2)	8.5% (Cole et al. 2012)[10]	Multiple linear regression, adjusted for parental BMI, parental educational attainment, child's sex and age at measurement and awake wearing time.	No association between sedentary behaviour, moderate PA, vigorous PA or MVPA and percentage body fat.
2) BODY MASS INDEX					
Longitudinal studies					
Butte et al. (2016)[3]	United States	n = 111, 58 boys, 53 girls; 3 - 5 years, 4.6(0.9)	18.0% (CDC)[4]	Mixed-effects linear models adjusted for age, sex, race/ethnicity, daycare hours, household size, household income, mother's age, BMI and education and awake time.	Children who spent more time engaged in MVPA had a larger change in BMI 1 year later. No relations between total PA or sedentary behaviour and BMI 1 year later.

Author (year)	Country and cohort	Participant characteristics (sample size; age[a])	Prevalence of overweight[b]	Statistical analysis	Conclusion
Jáuregui et al. (2012)[11]	Mexico	n = 205, 87 boys, 118 girls; 5 – 6, baseline 6.0(0.4), follow-up 8.1(0.3)	-	Multiple linear regression models adjusted by initial fat mass, energy intake and height, age, sex, socioeconomic status and changes in energy intake and height.	Children with a high baseline MVPA or who increased 10 min/d in MVPA showed no differences in BMI gain compared with their peers.
Leppänen et al. (2017)[5]	Sweden MINISTOP trial	n = 138, 73 boys, 65 girls; 4 years, 4.5(0.2) follow-up: 5.6(0.2)	7.2% baseline, 6.5% follow-up	Linear regression, adjusted for child's sex, age at measurement, ActiGraph awake wearing time.	Children who spent more time engaged in vigorous PA had a higher BMI 12 months later compared with their peers. No relation for sedentary behaviour, moderate PA or MVPA.
Metcalf et al. (2008)[12]	United Kingdom EarlyBird	n = 212, 113 boys, 99 girls; 5 – 8 years, follow up at 6, 7 and 8 years	-	Multiple linear regression.	No correlation between minutes in MVPA and changes in BMI.
Remmers et al. (2014)[13]	The Netherlands KOALA Birth Cohort	n = 297, 150 boys, 147 girls; T0: 4- 5 years, T1: 6-7, T2: 8-9 years.	-	GEE linear regression, adjusted for origin of BMI z-score, bicycling, swimming, season, recruitment group and paternal and maternal BMI.	A 5% increase in total PA or light PA resulted in decreased zBMI in heavier boys 1 year later, but not in leaner or normal weight boys or in girls. A 5% increase in MVPA resulted in decreased zBMI in normal weight and heavier boys and heavier girls 1 year later.
Cross-sectional studies					
Buck et al. (2015)[14]	Germany IDEFICS	n = 100, 57 boys, 43 girls; 2 - <6 years, 4.2(0.8)	-	Basic log-gamma regression model.	No association between BMI and MVPA.
Butte et al. (2016)[3]	United States	n = 111, 58 boys, 53 girls; 3 – 5 years, 4.6(0.9)	18.0% (CDC)[4]	Mixed-effects linear models adjusted for age, sex, race/ethnicity, daycare hours, household size, household income, mother's age, BMI and education and awake time.	No association between total PA, sedentary behaviour or MVPA and BMI.

Author (year)	Country and cohort	Participant characteristics (sample size; age[a])	Prevalence of overweight[b]	Statistical analysis	Conclusion
Byun et al. (2011)[15]	United States CHAMPS	n = 331, 168 boys, 163 girls; 2.8 – 5.7 years, 4.3(0.6)	-	Univariate analysis.	Girls who spent more time engaged in sedentary behaviour had a lower zBMI compared with their peers. No results for boys.
Cliff et al. (2009)[16]	Australia PANDA	n = 46, 25 boys, 21 girls; 3 – 5 years, 4.3(0.7)	-	Pearson product-moment correlations and Spearman rank-order correlations.	No association between total PA, sedentary behaviour, moderate PA, vigorous PA or MVPA and BMI.
Collings et al. (2017)[17]	United Kingdom BiB (HAPPY, BiB-1000, LEAP)	n = 333, 169 boys, 164 girls; 11 months – 5 years, 3.3(0.9)	19.5% (Cole et al. 1990)[18]	Multilevel models adjusted for age, sex, ethnicity, index of multiple deprivation, monitor worn time and season.	No association between total PA, sedentary behaviour, light PA or MVPA and BMI.
Dawson-Hahn et al. (2015)[19]	United States	n = 81, 47 boys, 34 girls; 3 - 5 years, 4.7(0.5)	-	Block linear regression analysis.	No association between BMI and total PA or MVPA.
España-Romero et al. (2013)[20]	United States SHAPES	n = 357, 183 boys, 174 girls; 3 – 5 years	Boys: 27.9%, girls: 28.7% (CDC)[4]	Linear mixed regression models adjusted for race/ethnicity, parental education and preschool.	Boys who spent more time engaged in MVPA had a higher zBMI compared with their peers. No results for girls and for sedentary behaviour.
Finn et al. (2002)[21]	United States	n = 214, 106 boys, 108 girls; 3 – 5 years, 3.9(0.1)	-	Forward-backward stepwise regression analysis.[d]	No association between total PA or vigorous PA and BMI.
Fisher et al. (2005)[22]	United Kingdom	n = 209, 101 boys, 108 girls; 4.8(1.2)	20.0% (UK 1990)[18]	Multiple regression analysis, including age, sex, zBMI and average temperature.	No association between zBMI and total PA.
Guo et al. (2017)[23]	United States CHAMPS	n = 227, 111 boys, 116 girls; 3 – 5 years, 4.2(0.6)	24.3% (CDC)[4]	Pearson correlations.	No association between total PA and zBMI.
Heelan and Eisenmann (2006)[7]	United States	n = 100, 48 boys, 52 girls; 4 – 7 years, 5.8(1.3)	-	Partial correlations controlling for chronological age.	No association between total PA or MVPA and BMI.

Author (year)	Country and cohort	Participant characteristics (sample size; age[a])	Prevalence of overweight[b]	Statistical analysis	Conclusion
Herzig et al. (2017)[24]	Switzerland SPLASHY	n = 309, 162 boys, 147 girls; 2 – 6 years, 3.9(0.7)	-	Pearson correlation coefficients.	No association between total PA or MVPA and BMI.
Iivonen et al. (2013)[25]	Finland	n = 37, 17 boys, 20 girls; 4 years, 4.1(0.3)	-	Multiple regression models for fundamental motor skills, adjusted for sex, age, BMI.	No associations between BMI and total PA or MVPA.
Jackson et al. (2003)[26]	United Kingdom SPARKLE	n = 104, 52 boys, 52 girls; 3 – 4 years, 3.7(0.4)	-	Correlation.	Children who spent more time engaged in total PA had a higher zBMI compared with their peers.
Kelly et al. (2006)[27]	United Kingdom MAGIC study	n = 339; 4.2(0.3)	-	Analysis of variance and covariance.	No association between zBMI and total PA.
Leppänen et al. (2016)[9]	Sweden MINISTOP trial	n = 307, 170 boys, 137 girls; 4.5(0.2)	8.5% (Cole et al. 2012)[10]	Multiple linear regression, adjusted for parental BMI, parental educational attainment, child's sex and age at measurement and awake wearing time.	No associations between sedentary behaviour, moderate PA, vigorous PA or MVPA and BMI.
Mendoza et al. (2014)[28]	United States	n = 96, 53 boys, 41 girls; 3 – 5 years, 4.7(0.5)	-	Block lineaer regression with age, sex, parent BMI and education, neighbourhood disorder, child acculturation, parent acculturation, tv viewing and MVPA added.	Children who spent more time engaged in MVPA had a lower zBMI compared with their peers.
Oliver et al. (2010)[29]	New Zealand	n = 78, 37 boys, 41 girls; 2 - 5 years	28.0% (IOTF)[2]	Univariable GEE regression for child PA rate.[d]	No association between total PA and BMI.

Author (year)	Country and cohort	Participant characteristics (sample size; age[a])	Prevalence of overweight[b]	Statistical analysis	Conclusion
Pfeiffer et al. (2009)[30]	United States CHAMPS	n = 331, 168 boys, 163 girls; 2.8 – 5.7 years, 4.3(0.6)	-	Pearson correlations.	Girls, and girls and boys together, who spent more time engaged in MVPA had a higher zBMI compared with their peers. No results for boys.
Schmutz et al. (2017)[31]	Switzerland SPLASHY	n = 394, 212 boys, 182 girls; 2 - 6 years, 3.9(0.7)	24.9% (WHO)[32]	Pearson correlation coefficient, received on request.	Children who spent more time engaged in total PA or MVPA and less time sedentary had a higher zBMI compared with their peers.
Toschke et al. (2007)[33]	Germany INCA	n = 192, 98 boys, 94 girls; 5 – 6 years	-	Pearson correlation.	No association between total PA and BMI.
Williams et al. (2008)[34]	United States CHAMPS	n = 198, 100 boys, 98 girls; 3 - 4 years, 4.2(0.5)	-	Bivariate correlations.	Children who spent more time engaged in MVPA had a higher zBMI compared with their peers. No results for sedentary behaviour, light PA or, vigorous PA.
Yamamoto et al. (2011)[35]	Germany Gesunde-Kinder	n = 645, 324 boys, 321 girls; 3 – 6 years	-	Multivariate model adjusted for age, parental education level, immigration status and siblings, parents' BMI, children's health and internal PA drive, daily television time and time spent outside, participation in organized sports, environmental opportunities and parents’ PA.	No association between BMI and MVPA.
3) WEIGHT STATUS[c]					
Cross-sectional studies					
Berglind et al. (2017)[36]	Sweden PRIMROSE trial	n = 540, 311 boys, 229 girls; 4 years, 4.2(0.2)	14.6% (Cole et al. 2000)[2]	Generalized estimating equation, adjusted for sex and maternal education.	No differences in total PA, sedentary behaviour, light PA or MVPA between children with and without overweight.

Author (year)	Country and cohort	Participant characteristics (sample size; age[a])	Prevalence of overweight[b]	Statistical analysis	Conclusion
Colley et al. (2013)[37]	Canada CHMS	n = 459, 232 boys, 227 girls; 3 – 5 years, 4.0(0.04)	16.4% (IOTF)[2]	-	No differences in total PA, sedentary behaviour, light PA or MVPA between children with and without overweight.
Ebenegger et al. (2012)[38]	Switzerland Ballabeina study	n = 600, 299 boys, 301 girls; 5.1(0.6)	20.0% (Swiss percentiles)[39]; 11.8% (IOTF)[2]	Linear regression analysis adjusted for sex, age and parental migrant status and educational level.	No differences in total PA, vigorous PA or MVPA between children with and without overweight.
Gutiérrez-Hervás et al. (2018)[40]	Spain	n = 136, 62 boys, 74 girls; 2 – 7 years, 5.5(1.5)	33.1% [41]	ANOVA.	Children without overweight spent more time engaged in total PA compared with children with overweight
Jones et al. (2009)[42]	Australia PANDA	n = 58; 2 – 6 years, 4.3(0.7)	19.6% (IOTF)[2]	Independent samples t-test.	No differences in total PA or MVPA between children with and without overweight.
Matarma et al. (2017)[43]	Finland STEPS study	n = 131, 58 boys, 73 girls; 5 years, 5.6(0.3)	16.8% [44]	Independent variables t-tests.	No differences in sedentary behaviour, light PA, moderate PA or vigorous PA between children with and without overweight. .
Matarma et al. (2018)[45]	Finland STEPS study	n = 111, 45 boys, 66 girls; 5 years, 5.6(0.4)	17.1% [44]	Independent variables t-tests.	No differences in sedentary behaviour or MVPA between children with and without overweight.
Metallinos-Katsaras et al. (2007)[46]	United States WIC	n = 56, 26 boys, 30 girls; 2 – 5 years	37.5% (CDC)[4]	Multiple linear regression adjusted for age, sex, race and monitor time worn.	No differences in light PA, moderate PA or vigorous PA between children with and without overweight.
Niederer et al. (2012)[47]	Switzerland	n = 613, 308 boys, 305 girls; 4 – 6 years	20.1% (Swiss national percentiles)[39]	ANCOVA adjusted for age-group, sex, preschool class (cluster).	No differences in total PA, vigorous PA or MVPA between 4, 5 and 6 year old children with and without overweight.

Author (year)	Country and cohort	Participant characteristics (sample size; age[a])	Prevalence of overweight[b]	Statistical analysis	Conclusion
O'Dwyer et al. (2011)[48]	United Kingdom Active Play	n = 50, 27 boys, 23 girls; 4.5(0.6)	boys: 26%, girls: 43% (Cole et al. 2000)[2]	Independent t-tests.	Boys without overweight spent more time engaged in moderate PA on weekdays compared with boys affected by overweight, but no differences for girls and on weekend days. No differences in sedentary behaviour, light PA, vigorous PA or MVPA between children with and without overweight.
Pate et al. (2015)[49]	United States CHAMPS, SHAPES	CHAMPS: n = 286, 122 boys, 164 girls; 3 - 5 years, 4.2(0.7)<br><br>SHAPES: n = 337, 173 boys, 164 girls; 3 - 5 years, 4.5(0.3)	CHAMPS: 28.3%, SHAPES: 28.5% (CDC)[4]	Analysis of variance with sex, race/ethnicity, parent education and weight status, adjusted for preschool.	No difference in total PA between children without overweight, children with overweight and children with obesity in CHAMPS as well as SHAPES.
Röttger et al. (2014)[50]	Germany, Switzerland, France	n = 114, 48 boys, 66 girls; 5.3(0.7)	17.9% (Percentiles German children)[51]	Independent t-test.	Children without overweight spent more time engaged in total PA compared with children with overweight, in both weekdays and weekend days.
Schaefer et al. (2015)[52]	United States NSFS	n = 134, 64 boys, 70 girls; 4 – 7 years, 5.6(1.0)	42.3 % (CDC)[4]	-[d]	Children without overweight spent less time engaged in sedentary behaviour compared with children with overweight. A trend was found in MVPA, where girls without overweight spent more time in MVPA compared with girls affected by overweight or obesity, no differences for boys.

Author (year)	Country and cohort	Participant characteristics (sample size; age[a])	Prevalence of overweight[b]	Statistical analysis	Conclusion
Tanaka and Tanaka (2013)[53]	Japan	n = 425, 223 boys, 202 girls; 5.8(0.6)	7.1% (Cole et al. 2000, 2007)[2,54]	ANCOVA adjusted for age and sex.	Thin children spent more time engaged in sedentary behaviour and less time in light PA and MVPA compared with children with and without overweight. Thin children spent more time engaged in vigorous PA compared with children without overweight, but not compared with children with overweight.
Trost et al. (2003)[55]	United States	n = 245, 118 boys, 127 girls; 3 – 5 years	Boys: 21.2%, girls 27.6% (CDC)[4]	ANCOVA adjusted for parent education.	Boys without overweight spent more time engaged in total PA, vigorous PA or MVPA compared with boys with overweight. No differences for girls.
Tucker et al. (2016)[56]	Canada LEAPP	n = 216, 102 boys, 114 girls; 2.5 – 5.9 years, 4.2(0.1)	24.5% (CDC)[4]	Three-way ANOVA with childcare as random factor.	No differences in total PA, vigorous PA or MVPA between children with and without overweight.
Vale et al. (2010)[57]	Portugal PRESTYLE	n = 281, 157 boys, 124 girls; 4 – 6, 5.0(0.8)	14.6% (≥ 1 SD)	Independent t-tests.	No differences in total PA, moderate PA or vigorous PA between children with and without overweight.
van Cauwenberghe et al. (2012)[58]	Australia HAPPY	n = 703, 387 boys, 316 girls; 3 - 5 years, 4.6(0.7)	17.5% (Cole et al. 2000)[2]	Multilevel logistic regression models.	No differences in hour by hour sedentary behaviour or MVPA patterns between children with and without overweight.
Vorwerg et al. (2013)[59]	Germany	n = 92, 51 boys, 40 girls; 3 – 6 years	14.0% (National percentiles German children)[51]	Wilcoxon test.	No difference in total PA between children with and without overweight.

4) WAIST CIRCUMFERENCE					
Longitudinal studies					
Author (year)	Country and cohort	Participant characteristics (sample size; age[a])	Prevalence of overweight[b]	Statistical analysis	Conclusion
Metcalf et al. (2008)[12]	United Kingdom EarlyBird	n = 212, 113 boys, 99 girls; 5 – 8 years, follow up at 6, 7 and 8 years	-	Multiple linear regression.	No correlation between minutes in MVPA and changes in waist circumference.
Cross-sectional studies					
Collings et al. (2017)[17]	United Kingdom BiB (HAPPY, BiB-1000, LEAP)	n = 333, 169 boys, 164 girls; 11 months – 5 years, 3.3(0.9)	19.5% (Cole et al. 1990)[18]	Multilevel models adjusted for age, sex, ethnicity, index of multiple deprivation, monitor worn time, season and height.	No association between total PA, sedentary behaviour, light PA or MVPA on waist circumference.
España-Romero et al. (2013)[20]	United States SHAPES	n = 357, 183 boys, 174 girls; 3 – 5 years	Boys: 27.9%, girls: 28.7% (CDC)[4]	Linear mixed regression models adjusted for race/ethnicity, parental education and preschool.	No association between sedentary behaviour or MVPA and waist circumference.
Leppänen et al. (2016)[9]	Sweden MINISTOP trial	n = 304, 169 boys, 135 girls; 4.5(0.2)	8.5% (Cole et al. 2012)[10]	Multiple linear regression, adjusted for parental BMI, parental educational attainment, child’s sex and age at measurement, awake wearing time and height.	No association between sedentary behaviour, moderate PA, vigorous PA or MVPA and waist circumference.
Oliver et al. (2010)[29]	New Zealand	n = 78, 37 boys, 41 girls; 2 – 5 years	28.0% (IOTF)[2]	Univariable GEE regression for child PA rate.	No association between total PA and waist circumference.
Oliver et al. (2013)[60]	New Zealand PIF:PAC	n = 126, 52 boys, 74 girls; 5.8 – 6.7, 5.9	58% high waist circumference	Multiple linear regression.	No association between sedentary behaviour or MVPA and waist circumference.

5) (TRUNK) FAT MASS (INDEX)					
Longitudinal studies					
Author (year)	Country and cohort	Participant characteristics (sample size; age[a])	Prevalence of overweight[b]	Statistical analysis	Conclusion
Butte et al. (2016)[3]	United States	n = 111, 58 boys, 53 girls; 3 - 5 years, 4.6(0.9)	18.0% (CDC)[4]	Mixed-effects linear models adjusted for age, sex, race/ethnicity, daycare hours, household size, household income, mother's age, BMI and education and awake time.	No relation between total PA, sedentary behaviour or MVPA and fat mass 1 year later.
Janz et al. (2009)[61]	United States IOWA-Fluoride study, IOWA-Bone Development study	n = 333, 148 boys, 185 girls; baseline: 5 years, follow-up: 8 and 11 years.	-	Mixed regression analysis adjusted for concurrent age, height, weight, fat mass at age 5 and MVPA.	MVPA at age 5 years was a predictor of later fat mass in boys, but not in girls.
Jáuregui et al. (2012)[11]	Mexico	n = 205, 87 boys, 118 girls; 5 – 6, baseline 6.0(0.4), follow-up 8.1(0.3)	-	Multiple linear regression models adjusted by initial fat mass, energy intake and height, age, sex, socioeconomic status and changes in energy intake and height.	Girls with a high baseline MVPA or girls who increased 10 min/d in MVPA had lower fat mass gain compared with their peers, but not for boys.
Leppänen et al. (2017)[5]	Sweden MINISTOP trial	n = 138, 73 boys, 65 girls; 4 years, 4.5(0.2) follow-up: 5.6(0.2)	7.2% baseline, 6.5% follow-up	Linear regression, adjusted for child's sex, age at measurement, ActiGraph awake wearing time.	No relation between sedentary behaviour, moderate PA, vigorous PA or MVPA and fat mass index 12 months later.
Cross-sectional studies					
Butte et al. (2016)[3]	United States	n = 111, 58 boys, 53 girls; 3 – 5 years, 4.6(0.9)	18.0% (CDC)[4]	Mixed-effects linear models adjusted for age, sex, race/ethnicity, daycare hours, household size, household income, mother's age, BMI and education and awake time.	No association between total PA, sedentary behaviour or MVPA and fat mass.

Author (year)	Country and cohort	Participant characteristics (sample size; age[a])	Prevalence of overweight[b]	Statistical analysis	Conclusion
Collings et al. (2013)[6]	United Kingdom SWS	n = 398, 202 boys, 196 girls; 4 years, 4.1(0.1)	20.1% (IOTF)[2]	Bivariate correlations.	Children who spent less time engaged in sedentary behaviour or more time in moderate PA, vigorous PA or MVPA had a lower fat mass index and trunk fat mass index compared with their peers. No results for light PA.
Heelan and Eisenmann (2006)[7]	United States	n = 100, 48 boys, 52 girls; 4 – 7 years, 5.8(1.3)	-	Partial correlations controlling for chronological age.	No association between total PA or MVPA and fat mass.
Jackson et al. (2009)[62]	United Kingdom RASCAL	n = 89, 42 boys, 47 girls; 2 – 6 years, 4.1 (1.3)	30.6% (UK 1990)[18]	General linear model, adjusted for age, sex and TV viewing time.	No association between fat mass and total PA.
Janz et al. (2002)[8]	United States IOWA-Fluoride study, IOWA-Bone Development study	n = 434, 203 boys, 231 girls; 4 – 6 years, 5.3(0.4)	-	Partial correlation coefficients adjusted for age and height.	Children who spent more time engaged in vigorous PA had a lower fat mass and trunk fat mass compared with their peers. Children who spent more time engaged in total PA had a lower fat mass compared with their peers. Girls who spent more time engaged in total PA had a lower trunk fat mass compared with their peers as well. No results for MVPA.
Kwon et al. (2011)[63]	United States IOWA-Bone Development study	n = 436, 204 boys, 232 girls; 5 years, 5.3(0.4)	-	Pearson partial correlation coefficients adjusted for age, height and fat free mass.	No associations between both IW-LPA100[e] and IW-LPA1100[e] and fat mass.
Leppänen et al. (2016)[9]	Sweden MINISTOP trial	n = 295, 166 boys, 129 girls; 4.5(0.2)	8.5% (Cole et al. 2012)[10]	Multiple linear regression, adjusted for parental BMI, parental educational attainment, child's sex and age at measurement and awake wearing time.	No association between sedentary behaviour, moderate PA, vigorous PA or MVPA and fat mass index.

6) SKINFOLD THICKNESS					
Longitudinal studies					
Author (year)	Country and cohort	Participant characteristics (sample size; age[a])	Prevalence of overweight[b]	Statistical analysis	Conclusion
Metcalf et al. (2008)[12]	United Kingdom EarlyBird	n = 212, 113 boys, 99 girls; 5 – 8 years, follow up at 6, 7 and 8 years	-	Multiple linear regression.	No correlation between minutes in MVPA and changes in skinfold thickness.
Cross-sectional studies					
Collings et al. (2017)[17]	United Kingdom BiB (HAPPY, BiB-1000, LEAP)	n = 333, 169 boys, 164 girls; 11 months – 5 years, 3.3(0.9)	19.5% (Cole et al. 1990)[18]	Multilevel models adjusted for age, sex, ethnicity, index of multiple deprivation, monitor worn time, season and height.	Children who spent more time engaged in MVPA had a lower skinfold thickness compared with their peers. No associations between total PA, sedentary behaviour or light PA and skinfold thickness.
Fang et al. (2017)[64]	China	n = 346, 201 boys, 145 girls; 3.5 – 5.5 years, 4.6(0.5)	-	multiple linear regression models controlling for age, BMI, sex and valid wearing time.	Boys who spent more time engaged in MVPA had a lower triceps skinfold thickness compared with their peers. No associations for girls or for light PA.
Herzig et al. (2017)[24]	Switzerland SPLASHY	n = 309, 162 boys, 147 girls; 2 – 6 years, 3.9(0.7)	-	Pearson correlation coefficients.	Children who spent more time engaged in total PA or MVPA had a lower sum of skinfolds compared with their peers.
7) OTHER ADIPOSITY OUTCOMES					
Mota et al. (2016)[65]	Portugal PRESTYLE	n = 646, 312 girls, 334 boys; 2 – 6 years, 5.2(0.7)	central obesity (WHtR > 0.5[f]); boys: 39.2%, girls: 52.4%	Logistic regression adjusted by age.	Boys classified as having central obesity spent more time in sedentary behaviour compared with boys who were not classified as central obesity. No statistical differences for girls.

Author (year)	Country and cohort	Participant characteristics (sample size; age[a])	Prevalence of overweight[b]	Statistical analysis	Conclusion
Said-Mohamed et al. (2012)[66]	Kameroen, central Afrika	n = 133; 2 – 6 years	24.7% stunted, 25.3% overweight, 13.6% stunted overweight[g]	ANCOVA, adjusted for sex, age and being at school.	Sedentary behaviour, light PA and MVPA were different between stunted children without overweight, stunted children with overweight, non-stunted children with overweight and non-stunted children without overweight.

Abbreviations: PA, physical activity; MVPA, moderate-to-vigorous physical activity; BMI, body mass index; IOTF, International Obesity Task Force age- and sex-specific BMI growth charts; CDC, Centers for Disease Control and Prevention; WHO, World Health Organization; SWS, Southampton Women's Survey; BiB, Born in Bradford study; CHMS, Canadian Health Measures Survey; WHtR, waist to height ratio.

[a] Age is presented as age range, mean(SD).

[b] Prevalence of overweight is presented as % overweight/obesity.

[c] Weight status was defined as non-overweight compared with overweight/obesity.

[d] Data received on request.

[e] IW-LPA100 is the daily sum of accelerometer counts during light-intensity physical activity defined as 100 - 2999 cpm, IW-LPA1100 is the daily sum of accelerometer counts during light-intensity physical activity defined as 1100 - 2999 cpm.

[f] Waist to height ratio was calculated as the ratio of waist (cm) and height (cm). A WHtR cutoff of <0.5 was used to define abdominal obesity (McCarthy and Ashwell, 2006). Two categories: the non-risk group (WHtR <0.5) and at risk group based on central fat (WHtR > 0.5).

[g] Stunted: height-for-age (HAC) ≤ 3rd percentile, BMI < 85th percentile, weight-for-height (WHZ) > -2 z-score; Overweight: HAC > 3rd percentile, BMI ≥ 85th percentile; Stunted Overweight: HAC ≤ 3rd percentile, BMI ≥ 85th percentile; Non-Stunted-and-Non-Overweight: HAC > 3rd percentile, BMI < 85th percentile, WHZ > -2 z-score.

References

1. Bürgi F, Meyer U, Granacher U, et al. Relationship of physical activity with motor skills, aerobic fitness and body fat in preschool children: a cross-sectional and longitudinal study (Ballabeina). *Int J Obes*. 2011;35(7):937-944. doi:10.1038/ijo.2011.54

2. Cole TJ, Bellizzi MC, Flegal KM, Dietz WH. Establishing a standard definition for child overweight and obesity worldwide: international survey. *BMJ*. 2000;320(7244):1240-1243. http://www.ncbi.nlm.nih.gov/pubmed/10797032. Accessed February 8, 2018.

3. Butte NF, Puyau MR, Wilson TA, et al. Role of physical activity and sleep duration in growth and body composition of preschool-aged children. *Obesity*. 2016;24(6):1328-1335. doi:10.1002/oby.21489

4. Kuczmarski RJ, Ogden CL, Grummer-Strawn LM, et al. CDC growth charts: United States. *Adv Data*. 2000;(314):1-27. http://www.ncbi.nlm.nih.gov/pubmed/11183293. Accessed February 8, 2018.

5. Leppänen MH, Henriksson P, Delisle Nyström C, et al. Longitudinal physical activity, body composition, and physical fitness in preschoolers. *Med Sci Sports Exerc*. 2017;49(10):2078-2085. doi:10.1249/MSS.0000000000001313

6. Collings PJ, Brage S, Ridgway CL, et al. Physical activity intensity, sedentary time, and body composition in preschoolers. *Am J Clin Nutr*. 2013;97(5):1020-1028. doi:10.3945/ajcn.112.045088

7. Heelan KA, Eisenmann JC. Physical Activity, Media Time, and Body Composition in Young Children. *J Phys Act Heal*. 2006;3(2):200-209. doi:10.1123/jpah.3.2.200

8. Janz KF, Levy SM, Burns TL, Torner JC, Willing MC, Warren JJ. Fatness, Physical Activity, and Television Viewing in Children during the Adiposity Rebound Period: The Iowa Bone Development Study. 2002;35(6):563-571. doi:10.1006/pmed.2002.1113

9. Leppänen MH, Nyström CD, Henriksson P, et al. Physical activity intensity, sedentary behavior, body composition and physical fitness in 4-year-old children: results from the ministop trial. *Int J Obes*. 2016;40(7):1126-1133. doi:10.1038/ijo.2016.54

10. Cole TJ, Lobstein T. Extended international (IOTF) body mass index cut-offs for thinness, overweight and obesity. *Pediatr Obes*. 2012;7(4):284-294. doi:10.1111/j.2047-6310.2012.00064.x

11. Jáuregui A, Villalpando S, Rangel-Baltazar E, Lara-Zamudio YA, Castillo-García MM. Physical activity and fat mass gain in Mexican school-age children: a cohort study. *BMC Pediatr*. 2012;12(1):620. doi:10.1186/1471-2431-12-109

12. Metcalf BS, Voss LD, Hosking J, Jeffery AN, Wilkin TJ. Physical activity at the government-recommended level and obesity-related

health outcomes: a longitudinal study (Early Bird 37). *Arch Dis Child*. 2008;93(9):772-777. doi:10.1136/adc.2007.135012

13. Remmers T, Sleddens EFC, Gubbels JS, et al. Relationship between Physical Activity and the Development of Body Mass Index in Children. *Med Sci Sport Exerc*. 2014;46(1):177-184. doi:10.1249/MSS.0b013e3182a36709

14. Buck C, Kneib T, Tkaczick T, Konstabel K, Pigeot I. Assessing opportunities for physical activity in the built environment of children: interrelation between kernel density and neighborhood scale. *Int J Health Geogr*. 2015;14(1):35. doi:10.1186/s12942-015-0027-3

15. Byun W, Dowda M, Pate RR. Correlates of Objectively Measured Sedentary Behavior in US Preschool Children. *Pediatrics*. 2011;128(5):937-945. doi:10.1542/peds.2011-0748

16. Cliff DP, Okely AD, Smith LM, Mckeen K. Relationships Between Fundamental Movement Skills and Objectively Measured Physical Activity in Preschool Children. *Pediatr Exerc Sci*. 2009;21:436-449.

17. Collings PJ, Brage S, Bingham DD, et al. Physical Activity, Sedentary Time, and Fatness in a Biethnic Sample of Young Children. *Med Sci Sport Exerc*. 2017;49(5):930-938. doi:10.1249/MSS.0000000000001180

18. Cole TJ, Freeman J V, Preece MA. Body mass index reference curves for the UK, 1990. *Arch Dis Child*. 1995;73(1):25-29. http://www.ncbi.nlm.nih.gov/pubmed/7639544. Accessed February 8, 2018.

19. Dawson-Hahn EE, Fesinmeyer MD, Mendoza JA. Correlates of Physical Activity in Latino Preschool Children Attending Head Start. *Pediatr Exerc Sci*. 2015;27(3):372-379. doi:10.1123/pes.2014-0144

20. España-Romero V, Mitchell JA, Dowda M, Neill JRO', Pate RR. Objectively Measured Sedentary Time, Physical Activity and Markers of Body Fat in Preschool Children. *Pediatr Exerc Sci*. 2013;25:154-163.

21. Finn K, Johannsen N, Specker B. Factors associated with physical activity in preschool children. *J Pediatr*. 2002;140(1):81-85. doi:10.1067/mpd.2002.120693

22. Fisher A, Reilly JJ, Montgomery C, et al. Seasonality in physical activity and sedentary behaviour in young children. *Pediatr Exerc Sci*. 2005;17:31-40. doi:10.1111/trf.13454

23. Guo H, Schenkelberg MA, O'Neill JR, Dowda M, Pate RR. How Does the Relationship Between Motor Skill Performance and Body Mass Index Impact Physical Activity in Preschool Children? *Pediatr Exerc Sci*. September 2017:1-19. doi:10.1123/pes.2017-0074

24. Herzig D, Eser P, Radtke T, et al. Relation of Heart Rate and its Variability during Sleep with Age, Physical Activity, and Body

Composition in Young Children. *Front Physiol*. 2017;8. doi:10.3389/fphys.2017.00109

25. Iivonen KS, Sääkslahti AK, Mehtälä A, et al. Relationship between Fundamental Motor Skills and Physical Activity in 4-Year-Old Preschool Children. *Percept Mot Skills*. 2013;117(2):627-646. doi:10.2466/10.06.PMS.117x22z7

26. Jackson DM, Reilly JJ, Kelly LA, Montgomery C, Grant S, Paton JY. Objectively Measured Physical Activity in a Representative Sample of 3- to 4-Year-Old Children. *Obes Res*. 2003;11(3):420-425. doi:10.1038/oby.2003.57

27. Kelly LA, Reilly JJ, Fisher A, et al. Effect of socioeconomic status on objectively measured physical activity. *Arch Dis Child*. 2006;91(1):35-38. doi:10.1136/adc.2005.080275

28. Mendoza JA, McLeod J, Chen T-A, Nicklas TA, Baranowski T. Correlates of Adiposity among Latino Preschool Children. *J Phys Act Heal*. 2014;11(1):195-198. doi:10.1123/jpah.2012-0018

29. Oliver M, Schofield GM, Schluter PJ. Parent influences on preschoolers' objectively assessed physical activity. *J Sci Med Sport*. 2010;13(4):403-409. doi:10.1016/j.jsams.2009.05.008

30. Pfeiffer KA, Dowda M, McIver KL, Pate RR. Factors Related to Objectively Measured Physical Activity in Preschool Children. *Pediatr Exerc Sci*. 2009;21:196-208. doi:10.1123/pes.21.2.196

31. Schmutz EA, Leeger-Aschmann CS, Radtke T, et al. Correlates of preschool children's objectively measured physical activity and sedentary behavior: A cross-sectional analysis of the SPLASHY study. *Int J Behav Nutr Phys Act*. 2017;14(1):1-13. doi:10.1186/s12966-016-0456-9

32. WHO Multicentre Growth Reference Study Group. WHO Child Growth Standards based on length/height, weight and age. *Acta Paediatr Suppl*. 2006;450:76-85. http://www.ncbi.nlm.nih.gov/pubmed/16817681. Accessed February 8, 2018.

33. Toschke JA, von Kries R, Rosenfeld E, Toschke AM. Reliability of physical activity measures from accelerometry among preschoolers in free-living conditions. *Clin Nutr*. 2007;26(4):416-420. doi:10.1016/j.clnu.2007.03.009

34. Williams HG, Pfeiffer KA, O'Neill JR, et al. Motor Skill Performance and Physical Activity in Preschool Children. *Obesity*. 2008;16(6):1421-1426. doi:10.1038/oby.2008.214

35. Yamamoto S, Becker S, Fischer J, De Bock F. Sex differences in the variables associated with objectively measured moderate-to-vigorous physical activity in preschoolers. *Prev Med (Baltim)*. 2011;52(2):126-129. doi:10.1016/j.ypmed.2010.11.014

36. Berglind D, Hansson L, Tynelius P, Rasmussen F. Levels and Patterns of Objectively Measured Physical Activity and Sedentary Time in 4-Year-Old Swedish Children. *J Phys Act Heal*. 2017;14(2):117-122. doi:10.1123/jpah.2016-0250

37. Colley RC, Garriguet D, Adamo KB, et al. Physical activity and sedentary behavior during the early years in Canada: a cross-sectional study. *Int J Behav Nutr Phys Act*. 2013;10:54-62. doi:10.1186/1479-5868-10-54

38. Ebenegger V, Marques-Vidal P, Kriemler S, et al. Differences in Aerobic Fitness and Lifestyle Characteristics in Preschoolers according to their Weight Status and Sports Club Participation. *Obes Facts*. 2012;5(1):23-33. doi:10.1159/000336603

39. Prader A, Largo RH, Molinari L, Issler C. Physical growth of Swiss children from birth to 20 years of age. First Zurich longitudinal study of growth and development. *Helv Paediatr Acta Suppl*. 1989;52:1-125. http://www.ncbi.nlm.nih.gov/pubmed/2737921. Accessed February 8, 2018.

40. Gutiérrez-Hervás A, Cortés-Castell E, Juste-Ruíz M, Palazón-Bru A, Gil-Guillén V, Rizo-Baeza M. Physical activity values in two-to-seven-year-old children measured by accelerometer over five consecutives 24-hour days. *Nutr Hosp*. 2018;35(3):252-257. doi:10.20960/nh.1403

41. World Health Organisation (WHO). Training course on child growth assessment. WHO Child Growth Standards. Module C - Interpreting Growth Indicators. http://www.who.int/childgrowth/training/c_interpretando.pdf. Published 2008.

42. Jones RA, Okely AD, Gregory P, Cliff DP. Relationships between weight status and child, parent and community characteristics in preschool children. *Int J Pediatr Obes*. 2009;4(1):54-60. doi:10.1080/17477160802199984

43. Matarma T, Tammelin T, Kulmala J, Koski P, Hurme S, Lagström H. Factors associated with objectively measured physical activity and sedentary time of 5–6-year-old children in the STEPS Study. *Early Child Dev Care*. 2017;187(12):1863-1873. doi:10.1080/03004430.2016.1193016

44. Saari A, Sankilampi U, Hannila M-L, Kiviniemi V, Kesseli K, Dunkel L. New Finnish growth references for children and adolescents aged 0 to 20 years: Length/height-for-age, weight-for-length/height, and body mass index-for-age. *Ann Med*. 2011;43(3):235-248. doi:10.3109/07853890.2010.515603

45. Matarma T, Lagström H, Hurme S, et al. Motor skills in association with physical activity, sedentary time, body fat, and day care attendance in 5–6-year-old children - the STEPS Study. *Scand J Med Sci Sports*. 2018:0-1. doi:10.1111/sms.13264

46. Metallinos-Katsaras ES, Freedson PS, Fulton JE, Sherry B. The Association Between an Objective Measure of Physical Activity and

Weight Status in Preschoolers. *Obesity*. 2007;15(3):686-694. doi:10.1038/oby.2007.571

47. Niederer I, Kriemler S, Zahner L, et al. BMI Group-Related Differences in Physical Fitness and Physical Activity in Preschool-Age Children. *Res Q Exerc Sport*. 2012;83(1):12-19. doi:10.1080/02701367.2012.10599820

48. O'Dwyer MV, Foweather L, Stratton G, Ridgers ND. Physical activity in non-overweight and overweight UK preschool children: Preliminary findings and methods of the Active Play Project. *Sci Sports*. 2011;26(6):345-349. doi:10.1016/j.scispo.2011.01.006

49. Pate RR, O'Neill JR, Brown WH, Pfeiffer KA, Dowda M, Addy CL. Prevalence of Compliance with a New Physical Activity Guideline for Preschool-Age Children. *Child Obes*. 2015;11(4):415-420. doi:10.1089/chi.2014.0143

50. Rottger K, Grimminger E, Kreuser F, Asslander L, Gollhofer A, Korsten-Reck U. Physical activity in different preschool settings. *J Obes*. 2014;2014(321701):1-8. doi:10.1155/2014/321701

51. Kromeyer-Hauschild K, Wabitsch M, Kunze D, et al. Perzentile für den Body-mass-Index für das Kindes- und Jugendalter unter Heranziehung verschiedener deutscher Stichproben. *Monatsschrift Kinderheilkd*. 2001;149(8):807-818. doi:10.1007/s001120170107

52. Schaefer SE, Camacho-Gomez R, Sadeghi B, Kaiser L, German JB, de la Torre A. Assessing Child Obesity and Physical Activity in a Hard-to-Reach Population in California's Central Valley, 2012–2013. *Prev Chronic Dis*. 2015;12:140577. doi:10.5888/pcd12.140577

53. Tanaka C, Tanaka S. Objectively-measured physical activity and body weight in Japanese pre-schoolers. *Ann Hum Biol*. 2013;40(6):541-546. doi:10.3109/03014460.2013.815802

54. Cole TJ, Flegal KM, Nicholls D, Jackson AA. Body mass index cut offs to define thinness in children and adolescents: international survey. *BMJ*. 2007;335(7612):194. doi:10.1136/bmj.39238.399444.55

55. Trost SG, Sirard JR, Dowda M, Pfeiffer KA, Pate RR. Physical activity in overweight and nonoverweight preschool children. *Int J Obes*. 2003;27(7):834-839. doi:10.1038/sj.ijo.0802311

56. Tucker P, Maltby AM, Burke SM, Vanderloo LM, Irwin JD. Comparing physical activity and sedentary time among overweight and nonoverweight preschoolers enrolled in early learning programs: a cross-sectional study. *Appl Physiol Nutr Metab*. 2016;41(9):971-976. doi:10.1139/apnm-2016-0021

57. Vale S, Silva P, Santos R, Soares-Miranda L, Mota J. Compliance with physical activity guidelines in preschool children. *J Sports Sci*. 2010;28(6):603-608. doi:10.1080/02640411003702694

58. Van Cauwenberghe E, Jones RA, Hinkley T, Crawford D, Okely AD. Patterns of physical activity and sedentary behaviour in preschool children. *Int J Behav Nutr Phys Act*. 2012;9:1-11. doi:10.1186/1479-5868-9-138

59. Vorwerg Y, Petroff D, Kiess W, Blüher S. Physical Activity in 3–6 Year Old Children Measured by SenseWear Pro®: Direct Accelerometry in the Course of the Week and Relation to Weight Status, Media Consumption, and Socioeconomic Factors. *PLoS One*. 2013;8(4):e60619. doi:10.1371/journal.pone.0060619

60. Oliver M, Schluter PJ, Healy GN, Tautolo E-S, Schofield G, Rush E. Associations Between Breaks in Sedentary Time and Body Size in Pacific Mothers and Their Children: Findings From the Pacific Islands Families Study. *J Phys Act Heal*. 2013;10:1166-1174.

61. Janz KF, Kwon S, Letuchy EM, et al. Sustained effect of early physical activity on body fat mass in older children. *Am J Prev Med*. 2009;37(1):35-40. doi:10.1016/j.amepre.2009.03.012

62. Jackson DM, Djafarian K, Stewart J, Speakman JR. Increased television viewing is associated with elevated body fatness but not with lower total energy expenditure in children. *Am J Clin Nutr*. 2009;89(4):1031-1036. doi:10.3945/ajcn.2008.26746

63. Kwon S, Janz KF, Burns TL, Levy SM. Association between Light-Intensity Physical Activity and Adiposity in Childhood. *Pediatr Exerc Sci*. 2011;23(2):218-229. doi:10.1123/pes.23.2.218

64. Fang H, Quan M, Zhou T, et al. Relationship between Physical Activity and Physical Fitness in Preschool Children: A Cross-Sectional Study. *Biomed Res Int*. 2017;2017:9314026. doi:10.1155/2017/9314026

65. Mota J, Silva Dos Santos S, Santos A, Seabra A, Vale S. Association between sedentary behavior time and waist-to-height ratio in preschool children. *Am J Hum Biol*. 2016;28(5):746-748. doi:10.1002/ajhb.22851

66. Said-Mohamed R, Bernard JY, Ndzana A-C, Pasquet P. Is Overweight in Stunted Preschool Children in Cameroon Related to Reductions in Fat Oxidation, Resting Energy Expenditure and Physical Activity? Johannsen D, ed. *PLoS One*. 2012;7(6):e39007. doi:10.1371/journal.pone.0039007

Appendix E. Physical activity assessment.

A) Studies using triaxial accelerometers

Author (year)	Type of accelerometer	Epoch (s)	Accelerometer cutpoints	Minimum weartime
Berglind et al. (2017)[1]	ActiGraph GT3X+	5	TPA: average cpm, SB: <820, LPA: 820 - 3907, MVPA: ≥ 3908 cpm[2]	At least 3 days, 10h/day
Iivonen et al. (2013)[3]	Actigraph GT3X	5	TPA: average cpm over monitoring period, MVPA: ≥ 196 counts/5s[4]	At least 3 days, 8h/day
Leppänen et al. (2016)[5]	ActiGraph wGT3x	10	SB <305, LPA 306-817, MPA 818-1968, VPA >1969, MVPA >818 VM (all per 5s)[6]	At least 3 days, 10h/day
Leppänen et al. (2017)[7]	ActiGraph wGT3x	10	SB <305, LPA 306-817, MPA 818-1968, VPA >1969, MVPA >818 VM (all per 5s)[6]	At least 3 days, 10h/day
Buck et al. (2015)[8]	ActiGraph GT3X+	15	MVPA = 2298 cpm[9]	At least 3 consecutive days, 1 weekend day, 8h/day
Collings et al. (2017)[10]	ActiGraph GT3X+	15	SB: <820, LPA: 820-3907, MVPA: ≥ 3904 cpm[2]	At least 1 valid day, 6h/day
España-Romero et al. (2013)[11]	ActiGraph GT1M and GT3X	15	SB: ≤ 200, MVPA: ≥ 420 counts/15s[12]	At least 2 weekdays, 6h/day
Matarma et al. (2017)[13]	ActiGraph GT3X	15	Cutpoints Evenson et al. (2008)[9]	At least 4 days, 8h/day, 3 weekdays and 1 weekend day
Matarma et al. (2018)[14]	ActiGraph GT3X	15	SB < 100cpm[9]	At least 4 days, 8h/day, 3 weekdays and 1 weekend day
Oliver et al. (2010)[15]	Actical accelerometer	15	TPA: daily PA rates	NR

Author (year)	Type of accelerometer	Epoch (s)	Accelerometer cutpoints	Minimum weartime
Pate et al. (2015)[16]	CHAMPS: ActiGraph 7164<br>SHAPES: GT1M and GT3X	15	TPA: ≥ 200 counts/15s[12]	At least 2 days, 8h/day
Schmutz et al. (2017)[17]	Actigraph wGT3X	15	TPA: mean cpm, SB: ≤ 25, MVPA: ≥ 420 counts/15s[9,12]	At least 3 days, 10h/day
Tucker et al. (2016)[18]	Actical accelerometer	15	TPA: ≥ 50, SB < 50, MVPA ≥ 715 counts/15s[19]	At least 3 days, 5h/day
Butte et al. (2016)[20]	ActiGraph GT3X+	60	SB: < 820, MVPA: ≥ 3908 cpm[2]	At least 4 valid days, 1 weekend day, 1000 min/day
Colley et al. (2013)[21]	Actical accelerometer	60	TPA: total cpm, SB: <100, LPA: 100 - 1149, MVPA: ≥ 1150 cpm[22,23]	At least 4 days, 5h/day
Jáuregui et al. (2012)[24]	RT3 accelerometers	60	MVPA: 970.2 cpm[25]	at least 1 valid day, 10h/day
Oliver et al. (2013)[26]	Actical accelerometer	60	SB: <100, MVPA: ≥1500 cpm[27]	At least 3 days, 7 hours/day
Tanaka and Tanaka (2013)[28]	ActivTracer GMS	60	Low-intensity activity (PAR < 2), LPA (2 < PAR < 3), MVPA (3 ≤ PAR) and VPA(PAR ≥ 4)[29]	At least 2 weekdays, 1 weekend day
Fang et al. (2017)[30]	Actigraph GT3X+	NR	LPA: 100-1679, MPA: 1680-3367, VPA ≥ 3368 cpm[12]	At least 3 days, 8h/day, 2 weekdays and 1 weekend day
Herzig et al. (2017)[31]	Actigraph wGT3x	NR	Cutpoints Butte et al (2014)[2]	At least 4 days, 10h/day
Röttger et al. (2014)[32]	AiperMotion 440	NR	SB: 4s resolution[33]	NR
Vorwerg et al. (2013)[34]	SensewarePro	NR	SB: MET ≤ 1.4, LPA: 1.5–2.9, MPA 3–5.9 , VPA ≥ 6 METs[35,36]	At least 4 consecutive days and nights, one day of the weekend

B) Studies using biaxial accelerometers

Author (year)	Type of accelerometer	Epoch (s)	Accelerometer cutpoints	Minimum weartime
Finn et al. (2002)[37]	Actiwatch Model AW16 activity monitor	NR	VPA: 1000 counts	NR

C) Studies using uniaxial accelerometers

Author (year)	Type of accelerometer	Epoch (s)	Accelerometer cutpoints	Minimum weartime
Mota et al. (2016)[38]	Actigraph GT1M	5	SB: ≤ 200 counts/15s[11,16]	At least 10h/day
O'Dwyer et al. (2011)[39]	Actigraph GT1M	5	Cupoints: Sirard et al. (2005)[40]	At least 3 days, 2 weekdays and 1 weekend
Vale et al. (2010)[41]	Actigraph GT1M	5	TPA: ≥ 1100, MPA: >1680, VPA: >3360 cpm[12,42]	At least 10h/day
Bürgi et al. (2011)[43]	Actigraph GT1M	15	MPA: 420 - 841, VPA: ≥ 842 counts/15s[12]	At least 3 days of recording, 2 weekdays and 1 weekend day, 6h/day
Byun et al. (2011)[44]	Actigraph 7164	15	SB: <37.5 counts/15s[12]	NR
Dawson-Hahn et al. (2015)[45]	Actigraph GT1M	15	TPA: > 37.5 cpm, MVPA: NR[12]	at least 5 days, 3h/day
Ebenegger et al. (2012)[46]	Actigraph GT1M	15	MVPA: ≥ 420, VPA: ≥ 842 counts/15s[12]	At least 3 days, 2weekdays and 1 weekend day, 6h/day
Guo et al. (2017)[47]	ActiGraph 7164	15	TPA: ≥ 200 counts/15s[11]	At least 3 days, 5 – 17 h/day
Mendoza et al. (2014)[48]	Actigraph GT1M	15	MVPA: ≥ 420 counts/15s[12]	At least 1 day, 8h/day
Niederer et al. (2012)[49]	Actigraph GT1M	15	TPA = total cpm, MVPA ≥ 420, VPA ≥ 842 epoch/h[12]	At least 3 days, 2weekdays and 1 weekend day, 6h/day
Pfeiffer et al. (2009)[50]	ActiGraph 7164	15	MVPA: ≥ 420 counts/15s[12]	At least 3 days
Remmers et al. (2014)[51]	ActiGraph 7164	15	SB : 0-25, LPA : 26-573, MPA : 574-1002, VPA : ≥ 1003 counts/epoch[9]	At least 3 days, 2weekdays and 1 weekend day, 400min/day

Author (year)	Type of accelerometer	Epoch (s)	Accelerometer cutpoints	Minimum weartime
Trost et al. (2003)[52]	ActiGraph 7164	15	MVPA: ≥ 3 MET's, VPA: ≥ 6 MET's[53]	At least 3 days
van Cauwenberghe et al. (2012)[54]	Actigraph GT1M	15	SB: ≤ 25, MVPA: 3yr. >614 | 4 yr. >811 | 5yr. > 890 counts/15s[9,40]	At least 4 days, 4 – 9h/day
Williams et al. (2008)[55]	ActiGraph 7164	15	SB: <37.5, LPA: 38-419, MVPA: ≥ 420, VPA: ≥ 842 counts/15s[12]	At least 3 days, 5h/day
Yamamoto et al. (2011)[56]	Actiheart	15	MVPA girls: > 105 | boys: > 118 counts/15s[57]	At least one weekend and one weekday
Schaefer et al. (2015)[58]	Polar Active	30	NR	at least 3 days
Cliff et al. (2009)[59]	Actigraph 7164	60	SB: <1100cpm, MPA: 3yr. 2460-4920 | 4yr. 3248-4936 | 5yr. 3564-5016, VPA: 3yr. >4920 | 4yr. >4936 | > 5016, MVPA: 3yr. > 2460 | 4yr. > 3248 | 5yr. > 3564 cpm[40,42]	At least three days, 6h/day
Collings et al. (2013)[21]	Actiheart combined heart rate and movement sensor	60	SB: < 37.5, LPA: 38 - 419, MPA: 420 - 841, VPA: >= 842 counts[12,50,55]	At least one valid day, 600 min/day
Fisher et al. (2005)[60]	CSA	60	TPA: average cpm over monitoring period[42,61]	At least 3 days, 6h/day
Heelan and Eisenmann (2006)[62]	ActiGraph 7164	60	MPA: 615-2971, VPA: > 2972 cpm[63]	At least 3 weekdays, 8h/day
Jackson et al. (2003)[64]	ActiGraph 7164/CSA	60	TPA: average over 3 day period	At least 3 days, 2weekdays, 1 weekend day, 6h/day
Jackson et al. (2009)[65]	Actiwatch-L	60	NR	NR

Author (year)	Type of accelerometer	Epoch (s)	Accelerometer cutpoints	Minimum weartime
Janz et al. (2002)[66]	CSA	60	TPA: total movement counts/total time, MVPA: ≥ 615, VPA: ≥ 2972 counts[63]	At least 3 days, minimal 8h/day
Janz et al. (2009)[67]	ActiGraph 7164	60	MVPA : > 3000 cpm[9,66,68–70]	At least 3 days, 8h/day
Jones et al. (2009)[71]	ActiGraph 7164	60	Total PA: mean cpm	At least 3 days, 6h/day
Kelly et al. (2006)[72]	Accelerometer	60	TPA: average cpm, SB: <1100, MVPA: 3200 cpm[64,73]	At least 6 days, 6h/day
Kwon et al. (2011)[74]	ActiGraph 7164	60	Daily sum of accelerometer counts at LPA (100 cpm) and LPA (1100 cpm)[42,75,76]	At least 3 days, 8h/day
Metallinos-Katsaras et al. (2007)[77]	ActiGraph 7164	60	LPA: <615, MPA: 615-2971, VPA: 2972-5331 cpm[78]	At least 4,5 days
Metcalf et al. (2008)[79]	Actigraph MTI and CSA	60	≥ 3 MET's[61,80]	NR
Said-Mohamed et al. (2012)[81]	Actigraph GT1M	60	SB: ≤ 800, LPA: ≤ 3200, MVPA: > 3201 cpm[61]	NR
Toschke et al. (2007)[82]	ActiGraph 7164	60	TPA: average active cpm	At least 6h/day
D) Type of accelerometer not mentioned				
Gutiérrez-Hervás et al. (2018)[83]	NR	15	TPA: total cpm	At least 4 days, 10 h/day, 3 weekdays and 1 weekend day

Abbreviations: TPA, total physical activity; SB, sedentary behaviour; LPA, light physical activity; MPA, moderate physical activity; VPA, vigorous physical activity; MVPA, moderate-to-vigorous physical activity; cpm, counts per minute; VM, vector magnitude; PAR, physical activity ratio; MET, metabolic equivalent; NR, not reported.

References

1. Berglind D, Tynelius P. Objectively measured physical activity patterns, sedentary time and parent-reported screen-time across the day in four-year-old Swedish children. *BMC Public Health*. 2017;18(1):1-9. doi:10.1186/s12889-017-4600-5

2. Butte NF, Wong WW, Lee JS, Adolph AL, Puyau MR, Zakeri IF. Prediction of energy expenditure and physical activity in preschoolers. *Med Sci Sports Exerc*. 2014;46(6):1216-1226. doi:10.1249/MSS.0000000000000209

3. Iivonen KS, Sääkslahti AK, Mehtälä A, et al. Relationship between Fundamental Motor Skills and Physical Activity in 4-Year-Old Preschool Children. *Percept Mot Skills*. 2013;117(2):627-646. doi:10.2466/10.06.PMS.117x22z7

4. van Cauwenberghe E, Labarque V, Trost SG, de Bourdeaudhuij I, Cardon G. Calibration and comparison of accelerometer cut points in preschool children. *Int J Pediatr Obes*. 2011;6(2-2):e582-e589. doi:10.3109/17477166.2010.526223

5. Leppänen MH, Nyström CD, Henriksson P, et al. Physical activity intensity, sedentary behavior, body composition and physical fitness in 4-year-old children: results from the ministop trial. *Int J Obes*. 2016;40(7):1126-1133. doi:10.1038/ijo.2016.54

6. Chandler JL, Brazendale K, Beets MW, Mealing BA. Classification of physical activity intensities using a wrist-worn accelerometer in 8-12-year-old children. *Pediatr Obes*. 2016;11(2):120-127. doi:10.1111/ijpo.12033

7. Leppänen MH, Henriksson P, Delisle Nyström C, et al. Longitudinal physical activity, body composition, and physical fitness in preschoolers. *Med Sci Sports Exerc*. 2017;49(10):2078-2085. doi:10.1249/MSS.0000000000001313

8. Buck C, Kneib T, Tkaczick T, Konstabel K, Pigeot I. Assessing opportunities for physical activity in the built environment of children: interrelation between kernel density and neighborhood scale. *Int J Health Geogr*. 2015;14(1):35. doi:10.1186/s12942-015-0027-3

9. Evenson KR, Catellier DJ, Gill K, Ondrak KS, McMurray RG. Calibration of two objective measures of physical activity for children. *J Sports Sci*. 2008;26(14):1557-1565. doi:10.1080/02640410802334196

10. Collings PJ, Brage S, Bingham DD, et al. Physical Activity, Sedentary Time, and Fatness in a Biethnic Sample of Young Children. *Med Sci Sport Exerc*. 2017;49(5):930-938. doi:10.1249/MSS.0000000000001180

11. España-Romero V, Mitchell JA, Dowda M, Neill JRO', Pate RR. Objectively Measured Sedentary Time, Physical Activity and Markers of Body Fat in Preschool Children. *Pediatr Exerc Sci*. 2013;25:154-163.

12. Pate RR, Almeida MJ, McIver KL, Pfeiffer KA, Dowda M. Validation and Calibration of an Accelerometer in Preschool Children*.

Obesity. 2006;14(11):2000-2006. doi:10.1038/oby.2006.234

13. Matarma T, Tammelin T, Kulmala J, Koski P, Hurme S, Lagström H. Factors associated with objectively measured physical activity and sedentary time of 5–6-year-old children in the STEPS Study. *Early Child Dev Care*. 2017;187(12):1863-1873. doi:10.1080/03004430.2016.1193016

14. Matarma T, Lagström H, Hurme S, et al. Motor skills in association with physical activity, sedentary time, body fat, and day care attendance in 5−6-year-old children - the STEPS Study. *Scand J Med Sci Sports*. 2018:0-1. doi:10.1111/sms.13264

15. Oliver M, Schofield GM, Schluter PJ. Parent influences on preschoolers' objectively assessed physical activity. *J Sci Med Sport*. 2010;13(4):403-409. doi:10.1016/j.jsams.2009.05.008

16. Pate RR, O'Neill JR, Brown WH, Pfeiffer KA, Dowda M, Addy CL. Prevalence of Compliance with a New Physical Activity Guideline for Preschool-Age Children. *Child Obes*. 2015;11(4):415-420. doi:10.1089/chi.2014.0143

17. Schmutz EA, Leeger-Aschmann CS, Radtke T, et al. Correlates of preschool children's objectively measured physical activity and sedentary behavior: A cross-sectional analysis of the SPLASHY study. *Int J Behav Nutr Phys Act*. 2017;14(1):1-13. doi:10.1186/s12966-016-0456-9

18. Tucker P, Maltby AM, Burke SM, Vanderloo LM, Irwin JD. Comparing physical activity and sedentary time among overweight and nonoverweight preschoolers enrolled in early learning programs: a cross-sectional study. *Appl Physiol Nutr Metab*. 2016;41(9):971-976. doi:10.1139/apnm-2016-0021

19. Pfeiffer KA, McIver KL, Dowda M, Almeida MJCA, Pate RR. Validation and calibration of the Actical accelerometer in preschool children. *Med Sci Sports Exerc*. 2006;38(1):152-157.

20. Butte NF, Puyau MR, Wilson TA, et al. Role of physical activity and sleep duration in growth and body composition of preschool-aged children. *Obesity*. 2016;24(6):1328-1335. doi:10.1002/oby.21489

21. Collings PJ, Brage S, Ridgway CL, et al. Physical activity intensity, sedentary time, and body composition in preschoolers. *Am J Clin Nutr*. 2013;97(5):1020-1028. doi:10.3945/ajcn.112.045088

22. Wong SL, Colley R, Gorber SC, Tremblay M. Actical Accelerometer Sedentary Activity Thresholds for Adults. *J Phys Act Heal*. 2011;8(4):587-591. doi:10.1123/jpah.8.4.587

23. Adolph AL, Puyau MR, Vohra FA, Nicklas TA, Zakeri IF, Butte NF. Validation of Uniaxial and Triaxial Accelerometers for the Assessment of Physical Activity in Preschool Children. *J Phys Act Heal*. 2012;9(7):944-953. doi:10.1123/jpah.9.7.944

24. Jáuregui A, Villalpando S, Rangel-Baltazar E, Lara-Zamudio YA, Castillo-García MM. Physical activity and fat mass gain in Mexican school-age children: a cohort study. *BMC Pediatr*. 2012;12(1):620. doi:10.1186/1471-2431-12-109

25. Rowlands A V, Thomas PWM, Eston RG, Topping R. Validation of the RT3 triaxial accelerometer for the assessment of physical activity. *Med Sci Sports Exerc*. 2004;36(3):518-524.

26. Oliver M, Schluter PJ, Healy GN, Tautolo E-S, Schofield G, Rush E. Associations Between Breaks in Sedentary Time and Body Size in Pacific Mothers and Their Children: Findings From the Pacific Islands Families Study. *J Phys Act Heal*. 2013;10:1166-1174.

27. Puyau MR, Adolph AL, Vohra FA, Zakeri I, Butte NF. Prediction of activity energy expenditure using accelerometers in children. *Med Sci Sports Exerc*. 2004;36(9):1625-1631.

28. Tanaka C, Tanaka S. Objectively-measured physical activity and body weight in Japanese pre-schoolers. *Ann Hum Biol*. 2013;40(6):541-546. doi:10.3109/03014460.2013.815802

29. Tanaka C, Tanaka S, Kawahara J, Midorikawa T. Triaxial Accelerometry for Assessment of Physical Activity in Young Children*. *Obesity*. 2007;15(5):1233-1241. doi:10.1038/oby.2007.145

30. Fang H, Quan M, Zhou T, et al. Relationship between Physical Activity and Physical Fitness in Preschool Children: A Cross-Sectional Study. *Biomed Res Int*. 2017;2017:9314026. doi:10.1155/2017/9314026

31. Herzig D, Eser P, Radtke T, et al. Relation of Heart Rate and its Variability during Sleep with Age, Physical Activity, and Body Composition in Young Children. *Front Physiol*. 2017;8. doi:10.3389/fphys.2017.00109

32. Rottger K, Grimminger E, Kreuser F, Asslander L, Gollhofer A, Korsten-Reck U. Physical activity in different preschool settings. *J Obes*. 2014;2014(321701):1-8. doi:10.1155/2014/321701

33. Kreuser F, Kromeyer-Hauschild K, Gollhofer A, Korsten-Reck U, Röttger K. “Obese equals Lazy?” analysis of the association between weight status and physical activity in children. *J Obes*. 2013;2013:437017.

34. Vorwerg Y, Petroff D, Kiess W, Blüher S. Physical Activity in 3–6 Year Old Children Measured by SenseWear Pro®: Direct Accelerometry in the Course of the Week and Relation to Weight Status, Media Consumption, and Socioeconomic Factors. *PLoS One*.

2013;8(4):e60619. doi:10.1371/journal.pone.0060619

35. Pate RR. Physical Activity Among Children Attending Preschools. *Pediatrics*. 2004;114(5):1258-1263. doi:10.1542/peds.2003-1088-L

36. Tanaka C, Tanaka S. Daily physical activity in japanese preschool children evaluated by triaxial accelerometry: the relationship between period of engagement in moderate-to-vigorous physical activity and daily step counts. *J Physiol Anthropol*. 2009;28(6):283-288.

37. Finn K, Johannsen N, Specker B. Factors associated with physical activity in preschool children. *J Pediatr*. 2002;140(1):81-85. doi:10.1067/mpd.2002.120693

38. Mota J, Silva Dos Santos S, Santos A, Seabra A, Vale S. Association between sedentary behavior time and waist-to-height ratio in preschool children. *Am J Hum Biol*. 2016;28(5):746-748. doi:10.1002/ajhb.22851

39. O'Dwyer MV, Foweather L, Stratton G, Ridgers ND. Physical activity in non-overweight and overweight UK preschool children: Preliminary findings and methods of the Active Play Project. *Sci Sports*. 2011;26(6):345-349. doi:10.1016/j.scispo.2011.01.006

40. Sirard JR, Trost SG, Pfeiffer KA, Dowda M, Pate RR. Calibration and evaluation of an objective measure of physical activity in preschool children. *J Phys Act Heal*. 2005;2(3):345.

41. Vale SMCG, Santos RMR, Soares-Miranda LM da C, Moreira CMM, Ruiz JR, Mota JAS. Objectively Measured Physical Activity and Body Mass Index in Preschool Children. *Int J Pediatr*. 2010;2010:1-6. doi:10.1155/2010/479439

42. Reilly JJ, Coyle J, Kelly L, Burke G, Grant S, Paton JY. An Objective Method for Measurement of Sedentary Behavior in 3- to 4-Year Olds. *Obes Res*. 2003;11(10):1155-1158. doi:10.1038/oby.2003.158

43. Bürgi F, Meyer U, Granacher U, et al. Relationship of physical activity with motor skills, aerobic fitness and body fat in preschool children: a cross-sectional and longitudinal study (Ballabeina). *Int J Obes*. 2011;35(7):937-944. doi:10.1038/ijo.2011.54

44. Byun W, Dowda M, Pate RR. Correlates of Objectively Measured Sedentary Behavior in US Preschool Children. *Pediatrics*. 2011;128(5):937-945. doi:10.1542/peds.2011-0748

45. Dawson-Hahn EE, Fesinmeyer MD, Mendoza JA. Correlates of Physical Activity in Latino Preschool Children Attending Head Start. *Pediatr Exerc Sci*. 2015;27(3):372-379. doi:10.1123/pes.2014-0144

46. Ebenegger V, Marques-Vidal P, Kriemler S, et al. Differences in Aerobic Fitness and Lifestyle Characteristics in Preschoolers according to their Weight Status and Sports Club Participation. *Obes Facts*. 2012;5(1):23-33. doi:10.1159/000336603

47. Guo H, Schenkelberg MA, O'Neill JR, Dowda M, Pate RR. How Does the Relationship Between Motor Skill Performance and Body Mass Index Impact Physical Activity in Preschool Children? *Pediatr Exerc Sci*. September 2017:1-19. doi:10.1123/pes.2017-0074

48. Mendoza JA, McLeod J, Chen T-A, Nicklas TA, Baranowski T. Correlates of Adiposity among Latino Preschool Children. *J Phys Act Heal*. 2014;11(1):195-198. doi:10.1123/jpah.2012-0018

49. Niederer I, Kriemler S, Zahner L, et al. BMI Group-Related Differences in Physical Fitness and Physical Activity in Preschool-Age Children. *Res Q Exerc Sport*. 2012;83(1):12-19. doi:10.1080/02701367.2012.10599820

50. Pfeiffer KA, Dowda M, McIver KL, Pate RR. Factors Related to Objectively Measured Physical Activity in Preschool Children. *Pediatr Exerc Sci*. 2009;21:196-208. doi:10.1123/pes.21.2.196

51. Remmers T, Sleddens EFC, Gubbels JS, et al. Relationship between Physical Activity and the Development of Body Mass Index in Children. *Med Sci Sport Exerc*. 2014;46(1):177-184. doi:10.1249/MSS.0b013e3182a36709

52. Trost SG, Sirard JR, Dowda M, Pfeiffer KA, Pate RR. Physical activity in overweight and nonoverweight preschool children. *Int J Obes*. 2003;27(7):834-839. doi:10.1038/sj.ijo.0802311

53. Sirard JS, Trost SG, Dowda M, Pate RR. Calibration of the computer science and applications, Inc. physical activity monitor in preschool children (Abstract). *Med Sci Sport Exerc*. 2001;5 (Suppl)(S144).

54. Van Cauwenberghe E, Jones RA, Hinkley T, Crawford D, Okely AD. Patterns of physical activity and sedentary behaviour in preschool children. *Int J Behav Nutr Phys Act*. 2012;9:1-11. doi:10.1186/1479-5868-9-138

55. Williams HG, Pfeiffer KA, O'Neill JR, et al. Motor Skill Performance and Physical Activity in Preschool Children. *Obesity*. 2008;16(6):1421-1426. doi:10.1038/oby.2008.214

56. Yamamoto S, Becker S, Fischer J, De Bock F. Sex differences in the variables associated with objectively measured moderate-to-vigorous physical activity in preschoolers. *Prev Med (Baltim)*. 2011;52(2):126-129. doi:10.1016/j.ypmed.2010.11.014

57. De Bock F, Menze J, Becker S, Litaker D, Fischer J, Seidel I. Combining accelerometry and HR for assessing preschoolers' physical activity. *Med Sci Sports Exerc*. 2010;42(12):2237-2243. doi:10.1249/MSS.0b013e3181e27b5d

58. Schaefer SE, Camacho-Gomez R, Sadeghi B, Kaiser L, German JB, de la Torre A. Assessing Child Obesity and Physical Activity in a Hard-to-Reach Population in California's Central Valley, 2012–2013. *Prev Chronic Dis*. 2015;12:140577. doi:10.5888/pcd12.140577

59. Cliff DP, Okely AD, Smith LM, Mckeen K. Relationships Between Fundamental Movement Skills and Objectively Measured Physical Activity in Preschool Children. *Pediatr Exerc Sci*. 2009;21:436-449.

60. Fisher A, Reilly JJ, Montgomery C, et al. Seasonality in physical activity and sedentary behaviour in young children. *Pediatr Exerc Sci*. 2005;17:31-40. doi:10.1111/trf.13454

61. Puyau MR, Adolph AL, Vohra FA, Butte NF. Validation and Calibration of Physical Activity Monitors in Children. *Obes Res*. 2002;10(3):150-157. doi:10.1038/oby.2002.24

62. Heelan KA, Eisenmann JC. Physical Activity, Media Time, and Body Composition in Young Children. *J Phys Act Heal*. 2006;3(2):200-209. doi:10.1123/jpah.3.2.200

63. Freedson PS, Sirard J, Debold E, et al. Calibration of the Computer Science and Applications, Inc. (CSA) accelerometer. *Med Sci Sport Exerc*. 1997;29(5):45.

64. Jackson DM, Reilly JJ, Kelly LA, Montgomery C, Grant S, Paton JY. Objectively Measured Physical Activity in a Representative Sample of 3- to 4-Year-Old Children. *Obes Res*. 2003;11(3):420-425. doi:10.1038/oby.2003.57

65. Jackson DM, Djafarian K, Stewart J, Speakman JR. Increased television viewing is associated with elevated body fatness but not with lower total energy expenditure in children. *Am J Clin Nutr*. 2009;89(4):1031-1036. doi:10.3945/ajcn.2008.26746

66. Janz KF, Levy SM, Burns TL, Torner JC, Willing MC, Warren JJ. Fatness, Physical Activity, and Television Viewing in Children during the Adiposity Rebound Period: The Iowa Bone Development Study. 2002;35(6):563-571. doi:10.1006/pmed.2002.1113

67. Janz KF, Kwon S, Letuchy EM, et al. Sustained effect of early physical activity on body fat mass in older children. *Am J Prev Med*. 2009;37(1):35-40. doi:10.1016/j.amepre.2009.03.012

68. Trost SG, Ward DS, Moorehead SM, Watson PD, Riner W, Burke JR. Validity of the computer science and applications (CSA) activity monitor in children. *Med Sci Sports Exerc*. 1998;30(4):629-633.

69. Trost SG, Way R, Okely AD. Predictive validity of three ActiGraph energy expenditure equations for children. *Med Sci Sports Exerc*. 2006;38(2):380-387. doi:10.1249/01.mss.0000183848.25845.e0

70. Jago R, Wedderkopp N, Kristensen PL, et al. Six-year change in youth physical activity and effect on fasting insulin and HOMA-IR. *Am J Prev Med*. 2008;35(6):554-560. doi:10.1016/j.amepre.2008.07.007

71. Jones RA, Okely AD, Gregory P, Cliff DP. Relationships between weight status and child, parent and community characteristics in preschool children. *Int J Pediatr Obes*. 2009;4(1):54-60. doi:10.1080/17477160802199984

72. Kelly LA, Reilly JJ, Fisher A, et al. Effect of socioeconomic status on objectively measured physical activity. *Arch Dis Child*. 2006;91(1):35-38. doi:10.1136/adc.2005.080275

73. Reilly JJ, Jackson DM, Montgomery C, et al. Total energy expenditure and physical activity in young Scottish children: mixed longitudinal study. *Lancet (London, England)*. 2004;363(9404):211-212.

74. Kwon S, Janz KF, Burns TL, Levy SM. Association between Light-Intensity Physical Activity and Adiposity in Childhood. *Pediatr Exerc Sci*. 2011;23(2):218-229. doi:10.1123/pes.23.2.218

75. Matthews CE, Chen KY, Freedson PS, et al. Amount of Time Spent in Sedentary Behaviors in the United States, 2003-2004. *Am J Epidemiol*. 2008;167(7):875-881. doi:10.1093/aje/kwm390

76. Treuth MS, Schmitz K, Catellier DJ, et al. Defining accelerometer thresholds for activity intensities in adolescent girls. *Med Sci Sports Exerc*. 2004;36(7):1259-1266.

77. Metallinos-Katsaras ES, Freedson PS, Fulton JE, Sherry B. The Association Between an Objective Measure of Physical Activity and Weight Status in Preschoolers. *Obesity*. 2007;15(3):686-694. doi:10.1038/oby.2007.571

78. Dowda M, Pate RR, Sallis J, Freedson PS. Accelerometer (CSA) Count Cut Points For Physical Activity Intensity Ranges In Youth. *Med Sci Sport Exerc*. 1997;29(5):72.

79. Metcalf BS, Voss LD, Hosking J, Jeffery AN, Wilkin TJ. Physical activity at the government-recommended level and obesity-related health outcomes: a longitudinal study (Early Bird 37). *Arch Dis Child*. 2008;93(9):772-777. doi:10.1136/adc.2007.135012

80. Schmitz KH, Treuth M, Hannan P, et al. Predicting energy expenditure from accelerometry counts in adolescent girls. *Med Sci Sports Exerc*. 2005;37(1):155-161.

81. Said-Mohamed R, Bernard JY, Ndzana A-C, Pasquet P. Is Overweight in Stunted Preschool Children in Cameroon Related to Reductions in Fat Oxidation, Resting Energy Expenditure and Physical Activity? Johannsen D, ed. *PLoS One*. 2012;7(6):e39007. doi:10.1371/journal.pone.0039007

82. Toschke JA, von Kries R, Rosenfeld E, Toschke AM. Reliability of physical activity measures from accelerometry among preschoolers in

free-living conditions. *Clin Nutr*. 2007;26(4):416-420. doi:10.1016/j.clnu.2007.03.009

83. Gutiérrez-Hervás A, Cortés-Castell E, Juste-Ruíz M, Palazón-Bru A, Gil-Guillén V, Rizo-Baeza M. Physical activity values in two-to-seven-year-old children measured by accelerometer over five consecutives 24-hour days. *Nutr Hosp*. 2018;35(3):252-257. doi:10.20960/nh.1403

Appendix F. Assessment of percentage of body fat, (trunk) fat mass (index), waist circumference, and skinfold thickness.

Author (year)	Adiposity outcome(s)	Measurement / cutpoints
Bürgi et al. (2011)[1]	Percentage of body fat	Four-polar single frequency bioelectric impedance.
Butte et al. (2016)[2]	Percentage of body fat<br>Fat mass	Dual energy X-ray absorptiometry.
Collings et al. (2013)[3]	Percentage of body fat<br>(Trunk) fat mass index	Dual energy X-ray absorptiometry.
Collings et al. (2017)[4]	Waist circumference<br>Skinfold thickness	Waist circumference: Seca anthropometrical tape at the level of the exposed naval.<br>Skinfold thickness: single measurements of triceps and subscapular skinfolds on the left side of the body using standard procedures.
España-Romero et al. (2013)[5]	Waist circumference	Tension-regulated tape, at midway between the inferior edge of the lowest rib and the superior border of iliac crest, at the end of a gentle expiration.
Fang et al. (2017)[6]	Skinfold thickness	Triceps skinfolds on the right side of the body.
Heelan and Eisenmann (2006)[7]	Percentage of body fat<br>Fat mass	Dual energy X-ray absorptiometry.
Herzig et al. (2017)[8]	Skinfold thickness	Triplicate measurements at four sites (biceps, triceps, subscapular, suprailiac) on the right body side.
Jackson et al. (2009)[9]	Fat mass	Dual energy X-ray absorptiometry.
Janz et al. (2002)[10]	Percentage of body fat<br>Fat mass	Dual energy X-ray absorptiometry.
Janz et al. (2009)[11]	Fat mass	Dual energy X-ray absorptiometry.
Jáuregui et al. (2012)[12]	Fat mass	Air-displacement plethysmography.
Kwon et al. (2011)[13]	Fat mass	Dual energy X-ray absorptiometry.
Leppänen et al. (2016)[14]	Percentage of body fat<br>Fat mass index<br>Waist circumference	Percentage of body fat and fat mass index: Air-displacement plethysmography.<br>Waist circumference: non-elastic tape (SECA model 200) at the umbilical location, at the end of a normal expiration.
Leppänen et al. (2017)[15]	Percentage of body fat<br>Fat mass index	Air-displacement plethysmography.
Metcalf et al. (2008)[16]	Waist circumference<br>Skinfold thickness	Waist circumference: NR.<br>Skinfold thickness: sum of biceps, triceps, subscapular, paraumbilical and suprailiac measurements.
Oliver et al. (2010)[17]	Waist circumference	Lufkin W606PM tape.
Oliver et al. (2013)[18]	Waist circumference	Lufkin W606PM tape, around the waist approximately half-way between the costal border and the iliac crest, with the participant breathing quietly.

Only the assessment of percentage of body fat, (trunk) fat mass (index), waist circumference, and skinfold thickness are shown. Body mass index was calculated as kg/m^2 in all included studies. The cut points for weight status can be found in Table 1.

References

1. Bürgi F, Meyer U, Granacher U, et al. Relationship of physical activity with motor skills, aerobic fitness and body fat in preschool children: a cross-sectional and longitudinal study (Ballabeina). *Int J Obes*. 2011;35(7):937-944. doi:10.1038/ijo.2011.54

2. Butte NF, Puyau MR, Wilson TA, et al. Role of physical activity and sleep duration in growth and body composition of preschool-aged children. *Obesity*. 2016;24(6):1328-1335. doi:10.1002/oby.21489

3. Collings PJ, Brage S, Ridgway CL, et al. Physical activity intensity, sedentary time, and body composition in preschoolers. *Am J Clin Nutr*. 2013;97(5):1020-1028. doi:10.3945/ajcn.112.045088

4. Collings PJ, Brage S, Bingham DD, et al. Physical Activity, Sedentary Time, and Fatness in a Biethnic Sample of Young Children. *Med Sci Sport Exerc*. 2017;49(5):930-938. doi:10.1249/MSS.0000000000001180

5. España-Romero V, Mitchell JA, Dowda M, O'Neill JR, Pate RR. Objectively Measured Sedentary Time, Physical Activity and Markers of Body Fat in Preschool Children. *Pediatr Exerc Sci*. 2013;25(1):154-163. doi:10.1123/pes.25.1.154

6. Fang H, Quan M, Zhou T, et al. Relationship between Physical Activity and Physical Fitness in Preschool Children: A Cross-Sectional Study. *Biomed Res Int*. 2017;2017:9314026. doi:10.1155/2017/9314026

7. Heelan KA, Eisenmann JC. Physical Activity, Media Time, and Body Composition in Young Children. *J Phys Act Heal*. 2006;3(2):200-209. doi:10.1123/jpah.3.2.200

8. Herzig D, Eser P, Radtke T, et al. Relation of Heart Rate and its Variability during Sleep with Age, Physical Activity, and Body Composition in Young Children. *Front Physiol*. 2017;8. doi:10.3389/fphys.2017.00109

9. Jackson DM, Djafarian K, Stewart J, Speakman JR. Increased television viewing is associated with elevated body fatness but not with lower total energy expenditure in children. *Am J Clin Nutr*. 2009;89(4):1031-1036. doi:10.3945/ajcn.2008.26746

10. Janz KF, Levy SM, Burns TL, Torner JC, Willing MC, Warren JJ. Fatness, Physical Activity, and Television Viewing in Children during the Adiposity Rebound Period: The Iowa Bone Development Study. 2002;35(6):563-571. doi:10.1006/pmed.2002.1113

11. Janz KF, Kwon S, Letuchy EM, et al. Sustained effect of early physical activity on body fat mass in older children. *Am J Prev Med*. 2009;37(1):35-40. doi:10.1016/j.amepre.2009.03.012

12. Jáuregui A, Villalpando S, Rangel-Baltazar E, Lara-Zamudio YA, Castillo-García MM. Physical activity and fat mass gain in Mexican school-age children: a cohort study. *BMC Pediatr*. 2012;12(1):620. doi:10.1186/1471-2431-12-109

13. Kwon S, Janz KF, Burns TL, Levy SM. Association between Light-Intensity Physical Activity and Adiposity in Childhood. *Pediatr Exerc Sci*. 2011;23(2):218-229. doi:10.1123/pes.23.2.218

14. Leppänen MH, Nyström CD, Henriksson P, et al. Physical activity intensity, sedentary behavior, body composition and physical fitness in 4-year-old children: results from the ministop trial. *Int J Obes*. 2016;40(7):1126-1133. doi:10.1038/ijo.2016.54

15. Leppänen MH, Henriksson P, Delisle Nyström C, et al. Longitudinal Physical Activity, Body Composition, and Physical Fitness in Preschoolers. *Med Sci Sports Exerc*. 2017;49(10):2078-2085. doi:10.1249/MSS.0000000000001313

16. Metcalf BS, Voss LD, Hosking J, Jeffery AN, Wilkin TJ. Physical activity at the government-recommended level and obesity-related health outcomes: a longitudinal study (Early Bird 37). *Arch Dis Child*. 2008;93(9):772-777. doi:10.1136/adc.2007.135012

17. Oliver M, Schofield GM, Schluter PJ. Parent influences on preschoolers’ objectively assessed physical activity. *J Sci Med Sport*. 2010;13(4):403-409. doi:10.1016/j.jsams.2009.05.008

18. Oliver M, Schluter PJ, Healy GN, Tautolo E-S, Schofield G, Rush E. Associations Between Breaks in Sedentary Time and Body Size in Pacific Mothers and Their Children: Findings From the Pacific Islands Families Study. *J Phys Act Heal*. 2013;10:1166-1174.

Appendix G. Overall and individual results of the risk of bias assessment using QUIPS.

I. Overall risk of bias scores for each QUIPS item, shown as a percentage of the total number of participants.

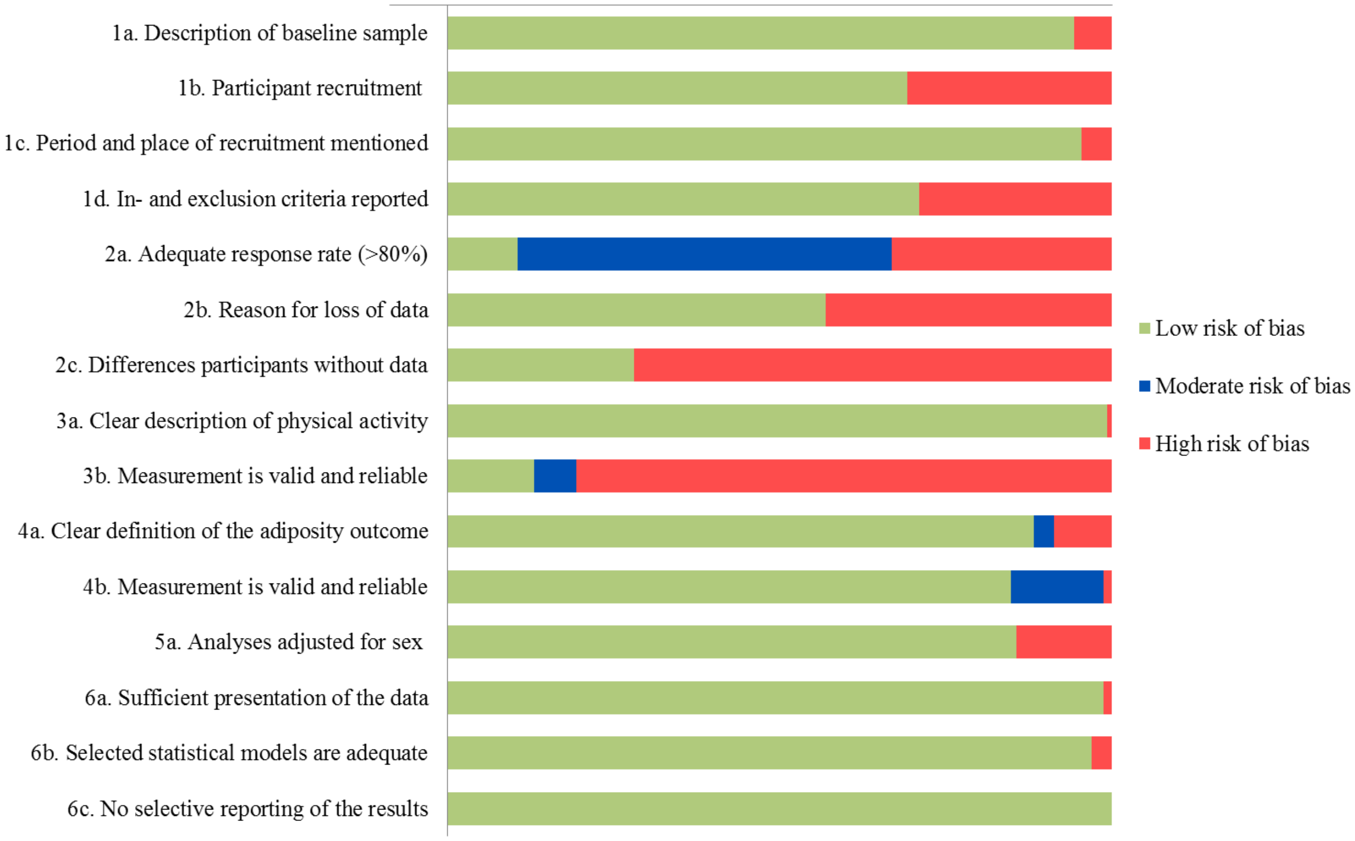

II. The results of the risk of bias assessment based on QUIPS for each study.

	**1. Participation**				**2. Attrition**			**3. Determinant**		**4. Outcome**		**5. Confounding**	**6. Analysis**		
Author, year	a. Description of baseline sample	b. Participant recruitment	c. Period and place of recruitment mentioned	d. In- and exclusion criteria reported	a. Adequate response rate (>80%)	b. Reason for loss of data	c. Differences participants without data	a. Clear description of physical activity	b. Measurement is valid and reliable	a. Clear definition of the adiposity outcome	b. Measurement is valid and reliable	a. Analyses adjusted for sex	a. Sufficient presentation of the data	b. Selected statistical models are adequate	c. No selective reporting of the results
Berglind et al. 2017	-	-	-	-	+	-	-	-	-	-	-	-	-	-	-
Buck et al. 2015	-	-	-	-	+/-	-	+	+	+	+	-	-	-	-	-
Bürgi et al. 2011	-	-	-	-	+	-	-	-	+	-	-	-	-	-	-
Butte et al. 2016	-	-	-	-	+	+	-	-	-	-	-	-	-	-	-
Byun et al. 2011	-	-	-	-	+/-	-	-	-	+	-	-	-	-	-	-
Cliff et al. 2009	-	-	-	+	+	-	-	-	+	-	-	-	-	-	-
Colley et al. 2013	-	-	-	-	+	-	+	-	+	+/-	+/-	+	-	+	-
Collings et al. 2013	-	-	-	-	+/-	-	+	-	+	-	-	-	-	-	-
Collings et al. 2017	-	-	-	-	+	-	-	-	+	-	-	-	-	-	-
Dawson-Hahn et al. 2015	-	-	-	-	+/-	-	+	-	+	-	-	-	-	-	-
Ebenegger et al. 2012	-	-	-	-	-	+	+	-	+	-	-	-	-	-	-
España-Romero et al. 2013	-	+	-	-	+/-	+	+	-	+	-	-	-	-	-	-
Fang et al. 2017	-	-	-	-	-	+	-	-	+	-	-	-	-	-	-
Finn et al. 2002	-	+	+	+	+/-	+	+	-	+	-	-	-	-	-	-
Fisher et al. 2005	-	-	-	-	+/-	-	+	-	+	-	-	-	-	-	-
Guo et al. 2017	-	-	-	-	+/-	+	+	-	+	-	-	-	-	-	-
Gutiérrez-Hervás et al. 2018	-	-	-	-	+	+	+	-	-	-	-	+	-	-	-
Heelan and Eisenmann 2006	-	-	-	+	+	-	+	-	+	-	-	-	-	-	-
Herzig et al. 2017	-	-	-	-	+/-	-	+	-	-	-	-	+	-	-	-
Iivonen et al. 2013	-	+	-	+	+	-	+	-	+	-	-	-	-	-	-
Jackson et al. 2003	-	-	-	-	+/-	-	+	-	+	-	-	+	-	-	-
Jackson et al. 2009	-	-	-	+	+/-	+	+	-	+/-	-	-	-	-	-	-
Janz et al. 2002	-	-	-	-	+	-	+	-	+	-	-	-	-	-	-
Janz et al. 2009	-	-	-	-	+/-	+	+	-	+	-	-	-	-	-	-
Jáuregui et al. 2012	-	-	-	-	+/-	-	-	-	+	-	-	-	-	-	-
Jones et al. 2009	-	-	-	+	+	+	+	-	+	-	-	+	-	-	-

II. The results of the risk of bias assessment based on QUIPS for each study (continued).

Author, year	a. Description of baseline sample	b. Participant recruitment	c. Period and place of recruitment mentioned	d. In- and exclusion criteria reported	a. Adequate response rate (>80%)	b. Reason for loss of data	c. Differences participants without data	a. Clear description of physical activity	b. Measurement is valid and reliable	a. Clear definition of the adiposity outcome	b. Measurement is valid and reliable	a. Analyses adjusted for sex	a. Sufficient presentation of the data	b. Selected statistical models are adequate	c. No selective reporting of the results
Kelly et al. 2006	-	-	-	+	+/-	+	+	-	+	+	+/-	-	-	-	-
Kwon et al. 2011	-	-	-	-	+/-	+	+	-	+	-	-	-	-	-	-
Leppänen et al. 2016	-	-	-	-	+/-	+	+	-	-	-	-	-	-	-	-
Leppänen et al. 2017	-	-	-	-	+	-	+	-	-	-	-	-	-	-	-
Matarma et al. 2017	-	-	-	-	+	+	+	-	+	-	-	+	-	-	-
Matarma et al. 2018	-	-	-	-	+	+	+	-	+	-	-	+	-	-	-
Mendoza et al. 2014	-	-	-	-	+/-	+	+	-	+	-	-	-	-	-	-
Metallinos-Katsaras et al. 2007	-	-	-	-	+	-	+	-	+/-	-	-	-	-	-	-
Metcalf et al. 2008	-	-	-	-	+/-	-	-	-	+	-	+/-	-	-	-	-
Mota et al. 2016	-	+	-	-	+/-	+	+	-	+	-	-	-	-	-	-
Niederer et al. 2012	-	+	-	+	-	-	+	-	+	-	-	-	-	-	-
O'Dwyer et al. 2011	-	+	-	+	+/-	+	+	-	+/-	-	-	-	-	-	-
Oliver et al. 2010	-	+	-	-	+	-	+	-	+/-	-	-	-	-	-	-
Oliver et al. 2013	-	-	-	-	+	-	-	-	+	-	-	-	-	-	-
Pate et al. 2015	-	+	-	+	+	+	-	-	+	-	-	-	-	-	-
Pfeiffer et al. 2009	-	+	-	+	+/-	+	+	-	+	-	-	-	-	-	-
Remmers et al. 2014	-	-	-	-	+/-	-	+	-	+	-	-	-	-	-	-
Röttger et al. 2014	-	-	+	+	+/-	-	+	-	+	-	-	+	-	-	-
Said-Mohammed et al. 2012	-	+	-	-	+	+	+	-	+	-	-	+	-	-	-
Schaefer et al. 2015	-	-	-	-	+/-	-	+	-	+/-	-	-	-	-	-	-
Schmutz et al. 2017	-	-	-	-	+/-	+	-	-	-	-	+/-	-	-	-	-
Tanaka and Tanaka 2013	-	-	-	-	+/-	-	+	-	+/-	-	-	-	-	-	-
Toschke et al. 2007	+	-	-	+	+/-	-	+	-	+	+	+	+	+	-	-
Trost et al. 2003	-	+	+	+	+/-	-	+	-	+	-	-	-	-	-	-
Tucker et al. 2016	-	-	-	-	+	-	+	-	+	-	-	-	-	-	-
Vale et al. 2010	-	+	-	+	+/-	+	+	-	+	-	-	+	-	-	-
Van Cauwenberghe et al. 2012	-	-	-	-	+	-	+	-	+	-	-	-	-	-	-
Vorwerg et al. 2013	-	+	+	+	+	-	+	-	+/-	-	-	+	-	-	-
Williams et al. 2008	-	+	-	+	+/-	-	+	-	+	-	-	-	-	-	-
Yamamoto et al. 2011	+	+	-	+	+/-	-	-	-	+	+	+/-	-	-	-	-

Appendix H. Forest plots of the association between physical activity and waist circumference, fat mass (index), and skinfold thickness, differentiated by physical activity intensities.

Total physical activity - waist circumference(cm)

Author (year)	Sex	Acc	Epoch	N	Point estimate	Standard error	p-Value
Collings (2017)	BG, adj	Tri	15	310	-0,017	0,037	0,644
Oliver (2010)	BG	Tri	15	78	-0,125	0,125	0,317
Overall					-0,026	0,035	0,467

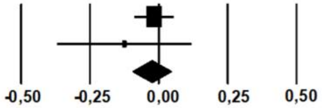

Heterogeneity: I^2=0%, Q=1, df=1, p=0.408

Sedentary behaviour - waist circumference(cm)

Author (year)	Sex	Acc	Epoch	N	Point estimate	Standard error	p-Value
España Romero (2013)	B	Tri	15	183	-0,100	0,075	0,179
España Romero (2013)	G	Tri	15	174	0,106	0,080	0,188
Collings (2017)	BG, adj	Tri	15	310	0,040	0,016	0,013
Lepännen (2016)	BG, adj	Tri	10	304	-0,407	0,339	0,230
Overall					0,012	0,049	0,813

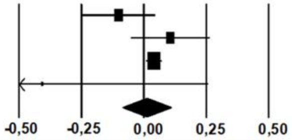

Heterogeneity: I^2=49%, Q=6, df=3, p=0.118

Moderate-to-vigorous physical activity - waist circumference(cm) *

Author (year)	Sex	Acc	Epoch	N	Point estimate	Standard error	p-Value
España Romero (2013)	B	Tri	15	183	0,109	0,075	0,145
España Romero (2013)	G	Tri	15	174	-0,079	0,078	0,310
Collings (2017)	BG, adj	Tri	15	310	-0,037	0,042	0,379
Lepännen (2016)	BG, adj	Tri	10	304	0,413	0,275	0,133

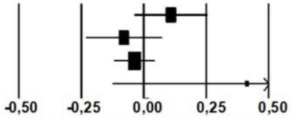

Heterogeneity: I^2=51%, Q=6, df=3, p=0.107
* The overall estimates are not shown because of heterogeneity.

Total physical activity - fat mass(kg)

Author (year)	Sex	Acc	Epoch	N	Point estimate	Standard error	p-Value
Heelan (2006)	B	Uni	60	48	-0,080	0,127	0,530
Janz (2002)	B	Uni	60	203	-0,150	0,077	0,050
Heelan (2006)	G	Uni	60	52	-0,130	0,207	0,530
Janz (2002)	G	Uni	60	231	-0,190	0,074	0,010
Butte (2016)	BG, adj	Tri	60	111	-0,083	0,083	0,317
Overall					-0,137	0,041	0,001

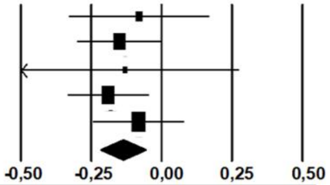

Heterogeneity: I^2=0%, Q=1, df=4, p=0.881

Moderate-to-vigorous physical activity - fat mass(kg)

Author (year)	Sex	Acc	Epoch	N	Point estimate	Standard error	p-Value
Heelan (2006)	B	Uni	60	48	-0,080	0,127	0,530
Janz (2002)	B	Uni	60	203	-0,070	0,111	0,530
Heelan (2006)	G	Uni	60	52	-0,220	0,350	0,530
Janz (2002)	G	Uni	60	231	-0,060	0,096	0,530
Butte (2016)	BG, adj	Tri	60	111	-0,164	0,087	0,061
Overall					-0,103	0,051	0,041

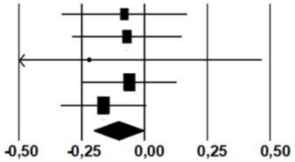

Heterogeneity: I^2=0%, Q=1, df=4, p=0.922

Sedentary behaviour - fat mass index(kg/m2)

Author (year)	Sex	Acc	Epoch	N	Point estimate	Standard error	p-Value
Collings (2013)	BG, adj	Uni	60	398	0,058	0,023	0,010
Lepännen (2016)	BG, adj	Tri	10	295	0,000	0,270	1,000
Overall					0,058	0,022	0,010

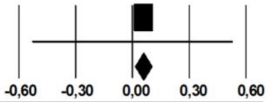

Heterogeneity: I^2=0%, Q=0, df=1, p=0.830

Moderate physical activity - fat mass index(kg/m2)

Author (year)	Sex	Acc	Epoch	N	Point estimate	Standard error	p-Value
Collings (2013)	BG, adj	Uni	60	398	-0,073	0,028	0,010
Lepännen (2016)	BG, adj	Tri	10	295	-0,245	0,367	0,504
Overall					-0,074	0,028	0,009

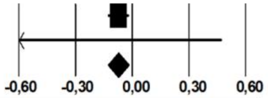

Heterogeneity: I^2=0%, Q=0, df=1, p=0.640

Vigorous physical activity - fat mass index(kg/m2)

Author (year)	Sex	Acc	Epoch	N	Point estimate	Standard error	p-Value
Collings (2013)	BG, adj	Uni	60	398	-0,120	0,036	0,001
Lepännen (2016)	BG, adj	Tri	10	295	-0,213	0,293	0,467
Overall					-0,121	0,036	0,001

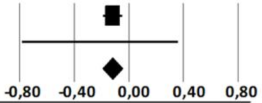

Heterogeneity: I^2=0%, Q=0, df=1, p=0.753

Appendix H. Forest plots of the association between physical activity and waist circumference, fat mass (index), and skinfold thickness, differentiated by physical activity intensities (continued).

Moderate-to-vigorous physical activity - fat mass index(kg/m2)

Author (year)	Sex	Acc	Epoch	N	Point estimate	Standard error	p-Value
Collings (2013)	BG, adj	Uni	60	398	-0,100	0,030	0,001
Lepännen (2016)	BG, adj	Tri	10	295	-0,274	0,274	0,317
Overall					-0,102	0,030	0,001

Point estimate and 95% CI

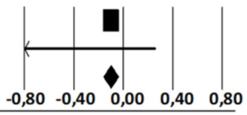

Heterogeneity: I^2=0%, Q=0, df=1, p=0.528

Total physical activity - skinfold thickness(mm)

Author (year)	Sex	Acc	Epoch	N	Point estimate	Standard error	p-Value
Collings (2017)	BG, adj	Tri	15	156	-0,145	0,094	0,123
Herzig (2017)	BG	Tri	NR	309	-0,120	0,061	0,050
Overall					-0,128	0,051	0,013

Point estimate and 95% CI

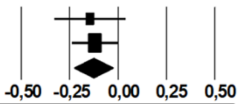

Heterogeneity: I^2=0%, Q=0, df=1, p=0.822

Light physical activity - skinfold thickness(mm)

Author (year)	Sex	Acc	Epoch	N	Point estimate	Standard error	p-Value
Fang (2017)	B	Tri	NR	201	0,026	0,041	0,530
Fang (2017)	G	Tri	NR	145	-0,077	0,123	0,530
Collings (2017)	BG, adj	Tri	15	156	-0,034	0,010	0,000
Overall					-0,029	0,013	0,030

Point estimate and 95% CI

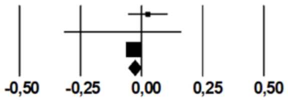

Heterogeneity: I^2=6%, Q=2, df=2, p=0.345

Moderate-to-vigorous physical activity - skinfold thickness(mm)

Author (year)	Sex	Acc	Epoch	N	Point estimate	Standard error	p-Value
Fang (2017)	B	Tri	NR	201	-0,195	0,099	0,050
Fang (2017)	G	Tri	NR	145	-0,041	0,065	0,530
Collings (2017)	BG, adj	Tri	15	156	-0,190	0,033	0,000
Herzig (2017)	BG	Tri	NR	309	-0,130	0,066	0,050
Overall					-0,145	0,036	0,000

Point estimate and 95% CI

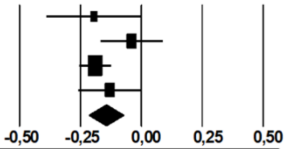

Heterogeneity: I^2=32%, Q=4, df=3, p=0.217

Abbreviations: B, boys; G, girls; BG, boys and girls; adj, adjusted for sex; acc, accelerometer type; uni, uniaxial; bi, biaxial; tri, triaxial; epoch, epoch length (s); N, number of participants.

Appendix I. The results of the subgroup analyses for waist circumference.[1]

		Q	df	p-value (Q)	I^2	N	stdβ	SE	p-value
Moderate-to-vigorous physical activity – waist circumference									
Sex	Boys	-	-	-	-	1	0.109	0.075	0.145
	Girls	-	-	-	-	1	-0.079	0.078	0.310
	Adjusted for sex	3	1	0.106	62%	-	-	-	-
Epoch length	10s	-	-	-	-	1	0.413	0.275	0.133
	15s	4	2	0.158	46%	3	-0.008	0.049	0.868
Prevalence of overweight[2]	Low prevalence	1	1	0.286	12%	2	0.146	0.099	0.141
	High prevalence	0.	1	0.629	0%	2	-0.046	0.037	0.210
Physical activity assessment[3]	Low risk	-	-	-	-	1	0.413	0.275	0.133
	Moderate/high risk	4	2	0.158	46%	3	-0.008	0.049	0.868
Missing data[3]	Low risk of bias	-	-	-	-	1	-0.037	0.042	0.379
	Moderate/high risk	5	2	0.082	60%	-	-	-	-

[1]*The results of the subgroup analyses are only shown if results were homogeneous.*

[2]*High prevalence of overweight was defined if >20% of the study sample was overweight/obese.*

[3]*Studies with low risk of bias on this QUIPS item compared to studies with a moderate/high risk of bias.*
